# Limited Transmission of Klebsiella pneumoniae among Humans, Animals, and the Environment in a Caribbean Island, Guadeloupe (French West Indies)

**DOI:** 10.1128/spectrum.01242-22

**Published:** 2022-09-12

**Authors:** Alexis Dereeper, Gaëlle Gruel, Matthieu Pot, David Couvin, Elodie Barbier, Sylvaine Bastian, Jean-Christophe Bambou, Moana Gelu-Simeon, Séverine Ferdinand, Stéphanie Guyomard-Rabenirina, Virginie Passet, Frederic Martino, Pascal Piveteau, Yann Reynaud, Carla Rodrigues, Pierre-Marie Roger, Xavier Roy, Antoine Talarmin, Benoit Tressieres, Marc Valette, Sylvain Brisse, Sébastien Breurec

**Affiliations:** a Transmission, Reservoir and Diversity of Pathogens Unit, Pasteur Institute of Guadeloupe, Pointe-à-Pitre, France; b UMR AgroEcologie, INRAE, Bourgogne Franche-Comté University, Dijon, France; c Laboratory of Clinical Microbiology, University Hospital Center of Guadeloupe, Pointe-à-Pitre/Les Abymes, France; d INRAE, ASSET, Petit-Bourg, France; e Hepato-Gastroenterology Department, University Hospital Center of Guadeloupe, Pointe-à-Pitre/Les Abymes, France; f Institut Pasteur grid.428999.7grid.452920.8 , University Paris Cité, Biodiversity and Epidemiology of Bacterial Pathogens, Paris, France; g Intensive Care Department, University Hospital Center of Guadeloupe, Pointe-à-Pitre/Les Abymes, France; h UR OPAALE, INRAE, Rennes, France; i Infectious Disease Department, University Hospital Center of Guadeloupe, Pointe-à-Pitre/Les Abymes, France; j Faculty of Medecine Hyacinthe Bastaraud, University of the Antilles, Pointe-à-Pitre, France; k Veterinary Clinic, Baie-Mahault, Guadeloupe; l INSERM Center for Clinical Investigation 1424, Pointe-à-Pitre/Les Abymes, France; Institute of Biomedical Sciences, Universidade de São Paulo

**Keywords:** *Klebsiella pneumoniae*, One Health, ESBL, genomic, Caribbean

## Abstract

Guadeloupe (French West Indies), a Caribbean island, is an ideal place to study the reservoirs of the Klebsiella pneumoniae species complex (KpSC) and identify the routes of transmission between human and nonhuman sources due to its insularity, small population size, and small area. Here, we report an analysis of 590 biological samples, 546 KpSC isolates, and 331 genome sequences collected between January 2018 and May 2019. The KpSC appears to be common whatever the source. Extended-spectrum-β-lactamase (ESBL)-producing isolates (21.4%) belonged to K. pneumoniae *sensu stricto* (phylogroup Kp1), and all but one were recovered from the hospital setting. The distribution of species and phylogroups across the different niches was clearly nonrandom, with a distinct separation of Kp1 and Klebsiella variicola (Kp3). The most frequent sequence types (STs) (≥5 isolates) were previously recognized as high-risk multidrug-resistant (MDR) clones, namely, ST17, ST307, ST11, ST147, ST152, and ST45. Only 8 out of the 63 STs (12.7%) associated with human isolates were also found in nonhuman sources. A total of 22 KpSC isolates were defined as hypervirulent: 15 associated with human infections (9.8% of all human isolates), 4 (8.9%) associated with dogs, and 3 (15%) associated with pigs. Most of the human isolates (33.3%) belonged to the globally successful sublineage CG23-I. ST86 was the only clone shared by a human and a nonhuman (dog) source. Our work shows the limited transmission of KpSC isolates between human and nonhuman sources and points to the hospital setting as a cornerstone of the spread of MDR clones and antibiotic resistance genes.

**IMPORTANCE** In this study, we characterized the presence and genomic features of isolates of the Klebsiella pneumoniae species complex (KpSC) from human and nonhuman sources in Guadeloupe (French West Indies) in order to identify the reservoirs and routes of transmission. This is the first study in an island environment, an ideal setting that limits the contribution of external imports. Our data showed the limited transmission of KpSC isolates between the different compartments. In contrast, we identified the hospital setting as the epicenter of antibiotic resistance due to the nosocomial spread of successful multidrug-resistant (MDR) K. pneumoniae clones and antibiotic resistance genes. Ecological barriers and/or limited exposure may restrict spread from the hospital setting to other reservoirs and vice versa. These results highlight the need for control strategies focused on health care centers, using genomic surveillance to limit the spread, particularly of high-risk clones, of this important group of MDR pathogens.

## INTRODUCTION

Klebsiella pneumoniae is currently recognized as an increasing threat to public health due to the emergence and spread of multidrug-resistant (MDR) isolates associated with hospital outbreaks. Recently, the WHO released a global priority list of antibiotic-resistant bacteria requiring new control strategies, including carbapenem-resistant K. pneumoniae (CRKP) and extended-spectrum-β-lactamase (ESBL)-producing K. pneumoniae (Kp-ESBL) isolates. ESBL and carbapenemase genes are located on mobile genetic elements and are frequently associated with genes encoding resistance to many other classes of antimicrobial agents, leading to bacteria that are difficult to treat. In Europe, it was estimated that 84,535 cases of infections with CRKP and Kp-ESBL bacteria occurred in 2015 and that these infections accounted for 5,805 attributable deaths (1). Estimations of the burden of MDR K. pneumoniae infections are lacking worldwide, but MDR rates are increasing globally. For example, in Senegal, K. pneumoniae isolates were the most common bacteria associated with neonatal bloodstream infections, of which 85% were ESBL producers (2). K. pneumoniae is also responsible for more severe invasive community-acquired infections, often in healthy individuals, including pyogenic liver abscess, pneumonia, and meningitis (3). Infections are caused by hypervirulent K. pneumoniae (HvKp) isolates, which belong to particular clonal groups (CGs). In the past, antimicrobial resistance (AMR) genes and virulence genes were present in specific nonoverlapping genomic lineages, but the frontiers are now blurring, resulting in the emergence of MDR and hypervirulent phenotypes in single K. pneumoniae isolates (4).

Phylogenetic studies have revealed that the former K. pneumoniae species is a genetically heterogeneous group. It has been redefined as the Klebsiella pneumoniae species complex (KpSC), a group of five closely related species distributed into seven phylogroups (Kp1 to Kp7): K. pneumoniae *sensu stricto* (phylogroup Kp1), K. quasipneumoniae subsp. *quasipneumoniae* (Kp2), *K. quasipneumoniae* subsp. *similipneumoniae* (Kp4), K. variicola subsp. *variicola* (Kp3), *K. variicola* subsp. *tropica* (Kp5), K. quasivariicola (Kp6), and K. africana (Kp7) (5). In this report, for simplicity, KpSC refers to the K. pneumoniae species complex, including the five species/seven phylogroups, and K. pneumoniae refers to K. pneumoniae *sensu stricto* (phylogroup Kp1).

In addition to their importance as human pathogens, members of the KpSC can be found in a wide range of ecological niches, such as soil, water, plants, insects, birds, reptiles, and the gut of many mammals (6). However, the prevalence and characteristics of KpSC isolates in these different niches are poorly known due to the lack of large dedicated research efforts. Despite the urgent public health threat now represented by the KpSC, knowledge of the dynamics of the transmission of this bacterial complex from environmental and animal reservoirs to humans using a broad ecological approach with whole-genome sequencing (WGS) is also scarce (7–11).

Guadeloupe, a tropical French overseas territory located in the Caribbean, is considered a very high-resource territory (https://hdr.undp.org). Data on the KpSC are scarce and recent for this island. An unusually high prevalence of HvKp (24%) was observed in adults admitted to the intensive care units of two university hospitals in Guadeloupe and Martinique (another French Caribbean territory) for spontaneous community-acquired bacterial meningitis (12). Guadeloupe also faced the emergence of hospital-acquired carbapenemase-producing K. pneumoniae infections (13) and an increased incidence of nosocomial Kp-ESBL infections (14, 15). Guadeloupe is an ideal place to study the reservoirs of KpSC isolates and identify the routes of transmission to the human population due to its insularity, small area (1,436 km^2^), and small population size (395,700 inhabitants in 2019). The primary objectives of our study were to determine the genomic features of a collection of isolated nonhuman K. pneumoniae strains regardless of the putative antibiotic resistance phenotype and to compare them with contemporaneous clinical isolates in order to define the reservoirs of clinical isolates and whether recent transmissions of this pathogen can be detected. The secondary objectives were to determine (i) the prevalence of KpSC members from different sources and (ii) their antibiotic susceptibility patterns.

## RESULTS

### KpSC isolates, phylogenetic diversity, and AMR.

For healthy food-producing animals, a total of 199 fecal samples from 124 pigs and 75 beef cattle were collected from 28 farms and the slaughterhouse (34 additional farms). The prevalences of KpSC isolates were 52.0% in bovines (39/75) and 24.2% in pigs (30/124). KpSC isolates were recovered from all nine poultry farms investigated (11 isolates).

For pets, a single rectal swab was taken from 149 dogs and 73 cats from the main animal shelter of Guadeloupe (*n* = 15) and 7 veterinary clinics (*n* = 170). For the identification of risk factors for the fecal carriage of ESBL-producing KpSC, 37 pets were eliminated because they displayed clinical signs of diarrhea and/or received antibiotic treatment in the previous month. Most of the pets were seen for vaccination (*n* = 79; 42.7%), surgery (*n* = 33; 17.8%), a preventive health visit (*n* = 28; 15.1%), or skin and soft tissue infection (*n* = 13; 7.0%). The rates of fecal carriage of KpSC were 27.4% (20/73) among cats and 49.0% (73/149) among dogs.

Dogs (*P* = 0.013), compared to cats, were significantly associated with KpSC fecal carriage (see Table S1 in the supplemental material).

Considering the environment, totals of 85 locally produced fresh vegetables (*n* = 45), flowering plants (*n* = 18), fruits (*n* = 11 [tomatoes only]), and aromatic herbs (*n* = 1 [thyme]) were collected. The prevalences of KpSC isolates were 90.1% (40/44) in vegetables, 66.6% (12/18) in flowering plants, and 36.4% (4/11) in fruits (tomatoes). The only aromatic herb tested was positive (thyme). Of the 44 raw water samples tested from 29 catchment points, KpSC isolates were detected in 15 samples (34.1%). Totals of 54 soil samples and 21 water samples from rivers or natural ponds located in proximity were investigated at 21 sites located throughout Guadeloupe. The frequencies of detection were 25.9% (14/54) and 33.3% (7/21), respectively. We did not find any significant association between the presence of KpSC isolates and the level of anthropic pressure (*P* = 0.795).

A total of 279 contemporaneous human KpSC isolates were recovered. These bacteria were isolated mainly from urine (*n* = 137; 49.1%), blood (*n* = 65; 23.3%), and wounds (*n* = 62; 22.2%). Six were associated with community liver abscess (10.5% of all community isolates), and 2 were associated with meningitis (3.5%). Of the 222 (79.6%) isolates associated with nosocomial infections, most of them were collected from patients hospitalized in medical wards (*n* = 110; 49.6%), intensive care units (*n* = 82; 36.9%), and emergency units (*n* = 62; 27.9%).

Low levels of resistance to antibiotics were observed for KpSC isolates whatever the source, except for those associated with hospital-acquired infections, which displayed significantly higher rates of resistance rates, whatever the antibiotic tested (*P* < 0.027). Full details of resistance to antibiotics according to the source are available in Table S2. All isolates with decreased phenotypic susceptibility to ertapenem were observed in the hospital setting (*n* = 10), 3 of which displayed carbapenemase genes (2 *bla*_KPC-2_ and 1 *bla*_NDM-1_). Kp-ESBL isolates (21.4%; 117/546) were associated with hospital-acquired infections only, except for 1 isolate recovered from the feces of one cat. The latter isolate also displayed resistance to fluoroquinolones. Resistance to this major family of antibiotics was not found in any isolate from other animals or the environment. All isolates collected from the environment were assigned a wild-type resistance phenotype, except for one isolate from a vegetable and two from soil (resistance to amoxicillin-clavulanic acid). Globally, the wild-type resistance phenotype corresponded to more than 80% of the isolates detected, except for the hospital setting, where this wild-type phenotype represented 31.8% (Table S2).

**FIG 1:**
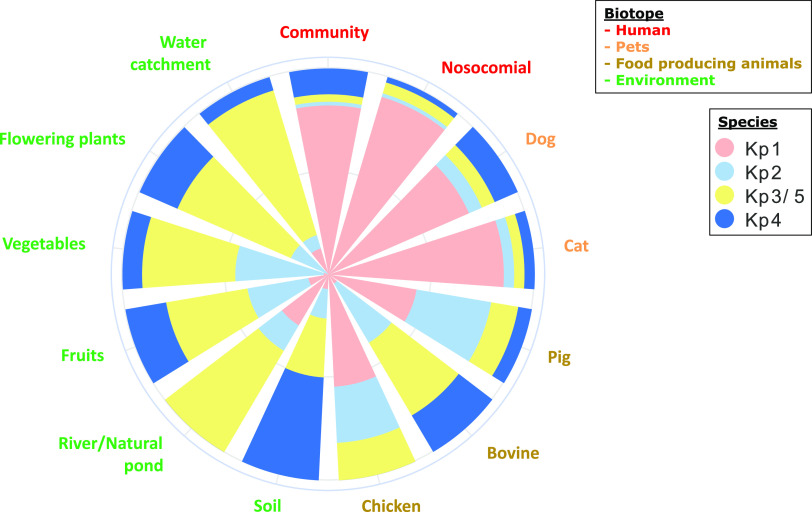
Species and phylogroup distributions of 433 isolates of the K. pneumoniae species complex collected in Guadeloupe (French West Indies), according to source. Species were defined by real-time PCR.

Phylogroup typing was performed by real-time PCR (RT-PCR) for all isolates except those recovered from the hospital setting, where ~50% were tested. The 433 KpSC isolates investigated were assigned to K. pneumoniae (Kp1 [*n* = 245; 56.6%], Kp2 [*n* = 62; 14.3%], Kp3 or Kp5 [*n* = 81; 18.7%], and Kp4 [*n* = 45; 10.4%]) (Fig. 1 and Table S3). Although the distribution of species and phylogroups across the various sources was clearly nonrandom, each phylogroup was isolated from all sources. A high prevalence of Kp1 and a low prevalence of Kp3 or Kp5 were observed in isolates associated with humans, domestic animals, pigs, and poultry, whereas the opposite relative frequencies of these groups were observed for vegetables, soil/rivers/natural ponds, catchment water, and bovines (Fig. 1 and Table S3). Kp4 was observed mainly in soil (*n* = 7; 50.0%), and Kp2 was observed mainly in food-producing animals (27.3% in poultry, 33.3% in bovines, and 36.7% in pigs) and on vegetables (45.2%). No significant difference was observed when comparing isolates from human and companion animals (*P* = 0.092), those from hospital and community settings (*P* = 0.09), and those from bovines and the environment (*P* = 0.79).

### Isolate genomic diversity.

**FIG 2:**
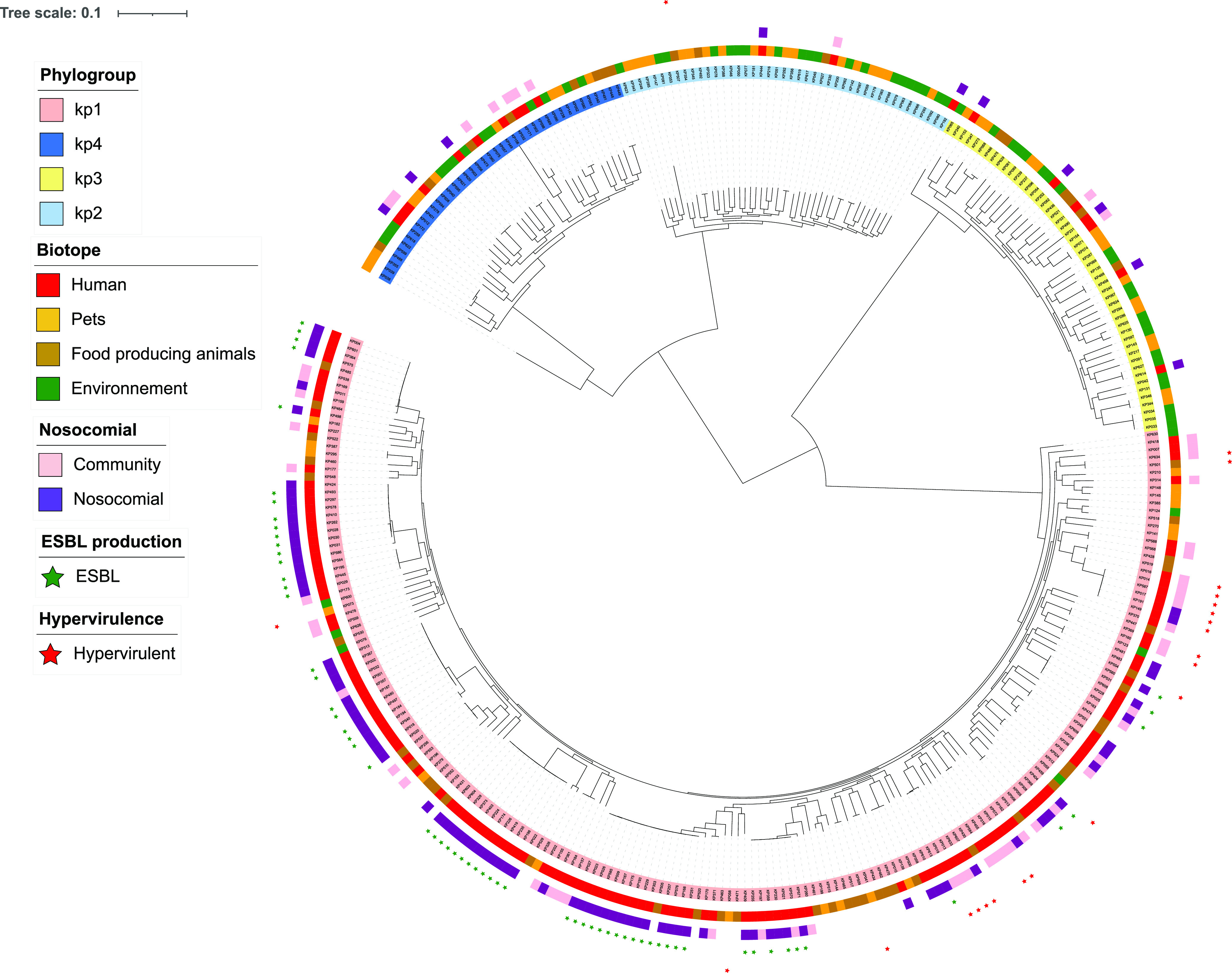
Phylogenetic tree of 331 isolates of the K. pneumoniae species complex from Guadeloupe (French West Indies). A maximum likelihood tree was constructed using RAxML based on the core-genome alignment and drawn with iTOL. Leaves are colored according to the phylogroup (Kp1, Kp2, Kp3, and Kp4), and annotation tracks are displayed as follows: source (human/animal/environment), nosocomial/community origin, ESBL production, and hypervirulence.

WGS was performed on a total of 331 isolates corresponding to a random selection of 66% of those collected from animals and the environment and on 55% of isolates associated with human infections. A full description of the isolates is displayed in Data Set S1 in the supplemental material. Phylogenetic analysis (Fig. 2) was performed to determine the frequencies of Kp1 (*n* = 196; 59.2%), Kp2 (*n* = 44; 13.3%), Kp3 (*n* = 50; 15.1%), and Kp4 (*n* = 41; 12.3%). Assignments were 100% concordant with the phylogrouping results for 433 isolates (see above).

High genetic diversity was found in all sources. A total of 218 sequence types (STs) were identified, with 163 being represented by a single isolate. Nineteen out of 218 STs were high-risk STs (90 isolates; 27.2% of the total), 17 of which were associated with MDR and 2 of which were associated with hypervirulence (ST23 and ST86) (Table 1). All STs represented by 5 or more isolates belonged to previously recognized successful clones. The most frequent STs were high-risk MDR clones, namely, ST17 (*n* = 16; 4.8%), ST307 (*n* = 12; 3.6%), ST11 (*n* = 11; 3.3%), ST147 (*n* = 9; 2.7%), ST152 (*n* = 8; 2.4%), and ST45 (*n* = 7; 2.1%). Most of them were human hospital-acquired Kp-ESBL isolates (47/63; 74.6%).

**TABLE 1:** High-risk clones (sequence types) collected from different sources in Guadeloupe (French West Indies)

Clone	No. of clones isolated from source				
	Humans	Cats	Dogs	Pigs	Total
Multidrug resistant					
ST11	11				11
ST13	4				4
ST14	2				2
ST15	1				1
ST17	14	2			16
ST20	2				2
ST29				1	1
ST36	1				1
ST37	1		3		4
ST39	1				1
ST45	6		1		7
ST101	1				1
ST147	9				9
ST152	8				8
ST258	2				2
ST307	12				12
ST392	1				1

Total	76	2	4	1	83
Hypervirulent					
ST23	5				5
ST86	1		1		2

Total	6		1		7

**FIG 3:**
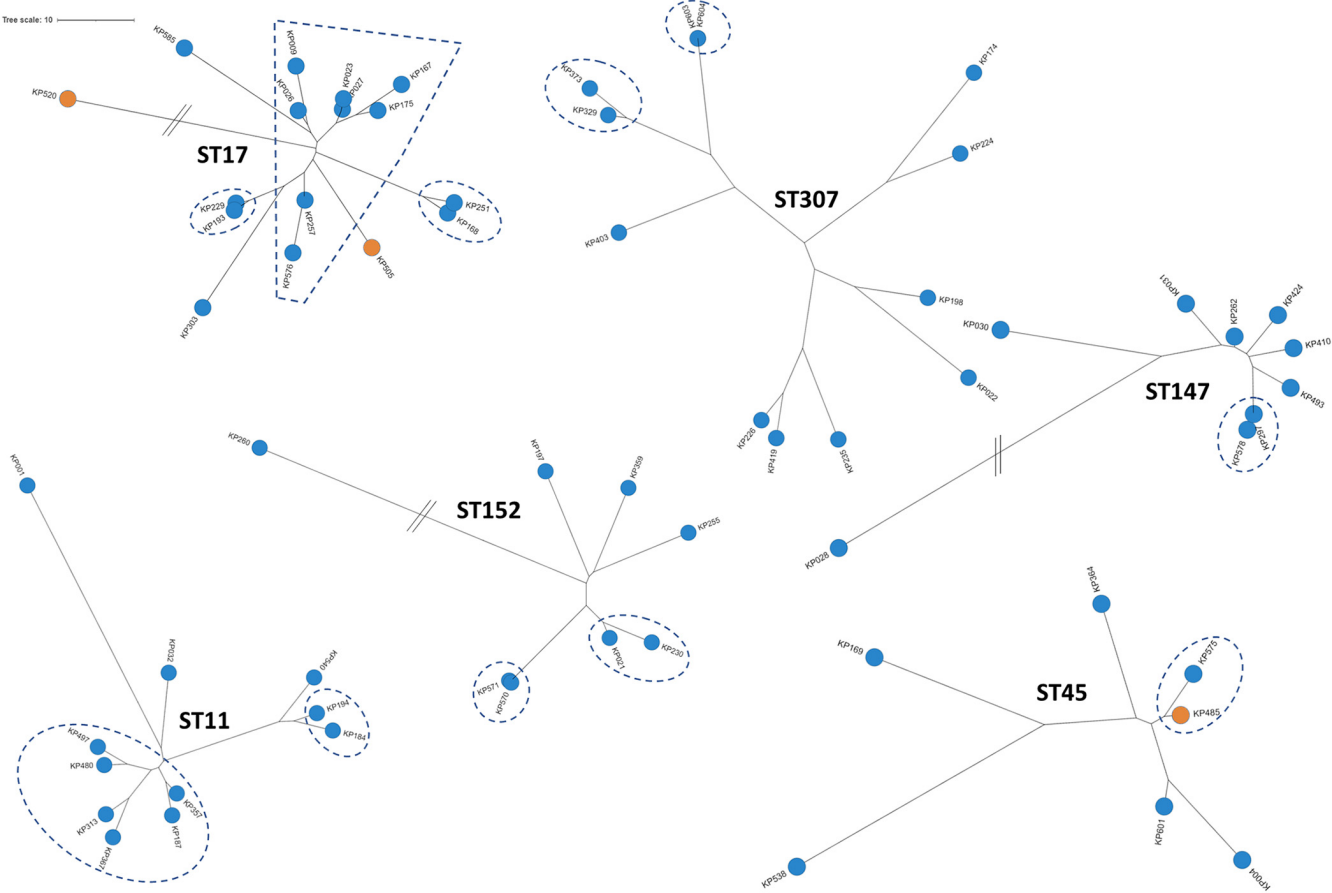
Unrooted phylogenetic trees of the genomes of the six most represented sequence types (STs) recovered in Guadeloupe (French West Indies). All these STs belong to K. pneumoniae *sensu stricto* (Kp1). Isolates are colored according to their source (i.e., human in blue and animal in orange). Using a threshold of <10 SNPs, single strains are framed with dotted lines.

Only eight STs (five belonged to K. pneumoniae, and three belonged to Kp3) were shared between nonhuman and human isolates, with six being strictly shared with pets: three high-risk MDR clones (ST17, ST37, and ST45), one high-risk hypervirulent clone (ST86), and four minor clones (ST2551, ST3600, ST4174, and ST5685). The frequency of STs from clinical isolates also detected in nonhuman samples was 12.7% (8/63). The two isolates assigned to ST45 were common to humans and dogs. They were almost identical (<10 core-genome multilocus sequence typing [cgMLST] allelic mismatches) and were isolated at 1-month intervals (Fig. 3 and Table S4). It should be noted that an ESBL gene (*bla*_CTX-M-15_) was recovered only in the human isolate. The other shared isolates were genetically more distant. Within the hospital setting, we observed the spread of closely related isolates (<10 single nucleotide polymorphisms [SNPs]) belonging to the four main STs (ST11, ST17, ST45, and ST307) during the 24-month period in different units of the hospital (Fig. 3 and Table S4). For the 4 main STs, isolates from Guadeloupe and those from other Caribbean islands (16) were not directly related based on their genomic sequences (Table S4).

### AMR genes and plasmids.

In agreement with the phenotypic antibiotic susceptibility patterns, a few isolates (7.9%; 14/178) with a gene(s) or mutation(s) conferring resistance to antimicrobials were recovered outside the human sources (see Table S5 in the supplemental material). Out of the 331 isolates investigated, 258 (77.9%) were classified into category 0 (low level of resistance). All of the remaining isolates (resistance scores of 1, 2, and 3 [*n* = 73]) belonged to Kp1 and were isolated mostly from humans (*n* = 69; 94.5%) and the hospital setting (*n* = 64; 87.7%). All isolates carrying an ESBL gene (*n* = 61; 18.4%) were recovered from human isolates associated with nosocomial infections, except for one collected from a cat, in agreement with the results of phenotypic antibiotic susceptibility testing. The ESBL genes were *bla*_CTX-M-15_ (*n* = 59) and *bla*_SHV-12_ (*n* = 3). One nosocomial isolate displayed *bla*_CTX-M-15_ and *bla*_SHV-12_. With regard to STs including ≥5 isolates, ST17 (15/16), ST147 (7/9), ST152 (5/8), and ST307 (12/12) had high absolute rates of 3rd-generation cephalosporin resistance (3GCR) of over 78%. MDR high-risk clones were associated with a high number of resistance genes, with 11 out of these 13 STs displaying a mean of 6 or more genes.

Two of the three carbapenemase-producing K. pneumoniae isolates were sequenced (Kp018 and Kp020); both harbored a *bla*_KPC-2_ gene, in agreement with PCR typing results (17). They were isolated in the hospital setting. These two isolates were closely related (25 cgMLST allelic mismatches, assigned to the common health care-associated clone ST258). They were classified into the highest-resistance category, harboring the carbapenemase gene *bla*_KPC-2_ and an alteration of the *mgrB* gene through total (Kp018) or partial (Kp020) deletion, known to confer colistin resistance. They were resistant to colistin (MIC > 4 mg/L). There was no other mechanism of colistin resistance detected in the other isolates, including the acquisition of the *mcr* gene.

Plasmid replicons were found in 67.3% (103/153) of human isolates, 67.8% (38/56) of isolates from pets, 48.3% (29/60) of isolates from food-producing animals, and 45.2% (28/62) of environmental isolates. IncFII (10.6%), IncFIA(HI1) (10.6%), ColRNAI (11.2%), IncR (53.0%), and IncFIB(K) (55.0%) were the most frequently recovered plasmid replicons, with IncFIA(HI1), IncFIB(K), IncFII, and IncR being present in all sources (Table S6).

### Virulence genes.

The prevalences of operons coding for acquired siderophores were 24.2% for yersiniabactin (80/331; 3/80 were incomplete), 6.0% for aerobactin (20/331), and 5.7% for salmochelin (19/331). The genotoxin colibactin loci were present in 4.5% (15/331; 1/15 was incomplete) of the isolates (see Table S7 in the supplemental material). Virulence genes were recovered mainly in human and pet isolates. By comparing human and pet isolates, yersiniabactin (43.8% versus 17.9% [*P* = 0.001]) and colibactin (9.8% versus 0% [*P* = 0.013]) frequencies were significantly higher in humans, but no significant difference was observed for aerobactin (9.2% versus 7.1% [*P* = 0.785]) and salmochelin (5.2% versus 1.9% [*P* = 0.450]). Five isolates from pigs displayed virulence genes, two with yersiniabactin and three with aerobactin, as did one from soil (yersiniabactin). The three aerobactin-positive isolates from pigs carried an *iuc3* aerobactin gene. They came from different farms and belonged to three different genetic backgrounds (ST29, ST432, and ST827). In addition, the *iuc3* gene was recovered on a contig predicted to be plasmidic by combining the MOB-recon and PlasFlow tools. For two out of the three isolates, *iuc3* and an IncFIB(K) replicon were present in the same contig. It should be noted that a presumed plasmid carrying *iuc3* was recovered in one isolate from a dog. The isolate carried an IncFIB(K) replicon but was not recovered on the *iuc3*-positive contig.

In all, a total of 22 isolates (6.6%) were defined as hypervirulent: 15 were associated with community (*n* = 12; 21.4%) and nosocomial (*n* = 3; 3.1%) human infections, 4 (8.9%) were associated with dogs, and 3 (15%) were associated with pigs. The genomic features of HvKp isolates are displayed in Table 2. ST23 (subclade I) was the ST most frequently recovered in human isolates (33.3% of human HvKp isolates). Nine isolates (34.6%) harbored the regulator of mucoid phenotype gene *rmpA*, whereas yersiniabactin, colibactin, and aerobactin operons were present together in 11 (50.0%) isolates. All isolates belonged to K. pneumoniae, except for one Kp2 isolate associated with fecal carriage in a dog. These hypervirulence-associated operons were observed mostly in isolates associated with human infections (15/22; 68.2%). The remaining hypervirulent isolates were associated with fecal carriage in dogs (*n* = 4) and pigs (*n* = 3). Human infections corresponded mostly to community-acquired infections (12/15; 80%) and were associated mostly with liver abscess (*n* = 5) and meningitis (*n* = 2). The human hypervirulent isolates were associated mostly with STs typically associated with capsular serotypes K1 (*n* = 5; 22.7%) and K2 (*n* = 9; 40.9%), with the most frequently recovered genetic backgrounds being ST23 (*n* = 5), ST66 (*n* = 2), ST86 (*n* = 2), ST380 (*n* = 2), and ST3253 (*n* = 2). ST23 isolates belonged to the CG23-I lineage (data not shown) according to Lam et al. (18) and carried a yersiniabactin-encoding mobile element (ICEKp10), including genes coding for yersiniabactin and colibactin. All HvKp isolates exhibited the wild-type AMR phenotype.

**TABLE 2:** Genomic features of 22 hypervirulent K. pneumoniae species complex isolates collected in Guadeloupe (French West Indies) according to source^*a*^

Feature	No. (%) of isolates with feature				
	Humans		Dogs (*n* = 4)	Pigs (*n* = 3)	Total (*n* = 22)
	Community (*n* = 12)	Nosocomial (*n* = 3)			
Phylogroup					
K. pneumoniae (Kp1)	12 (100)	3 (100)	3 (75)	3 (100)	21 (95.5)
*K. quasipneumoniae* subsp. *quasipneumoniae* (Kp2)	0 (0)	0 (0)	1 (25)	0 (0)	1 (4.5)
Virulence gene					
Colibactin (*clb*)	10 (83.3)	2 (66.7)	0 (0)	0 (0)	12 (54.5)
Aerobactin (*iuc*)	12 (100)	3 (100)	2 (50)	3 (100)	20 (90.9)
Salmochelin (*iro*)	12 (100)	3 (100)	4 (100)	0 (0)	19 (86.4)
*rmpA*	5 (41.7)	2 (66.7)	2 (50)	0 (0)	9 (40.9)
*rmpA2*	0 (0)	0 (0)	1 (25)	0 (0)	1 (4.5)
CG and associated ST					
CG5 ST5	0 (0)	0 (0)	1 (25)	0 (0)	1 (4.5)
CG23 ST23	3 (25)	2 (66.7)	0 (0)	0 (0)	5 (22.7)
CG29 ST29	0 (0)	0 (0)	0 (0)	1 (33.3)	1 (4.5)
CG35 ST5750	0 (0)	1 (33.3)	0 (0)	0 (0)	1 (4.5)
CG60 ST60	0 (0)	0 (0)	1 (25)	0 (0)	1 (4.5)
CG65 ST3253	2 (16.7)	0 (0)	0 (0)	0 (0)	2 (9.1)
CG66 ST66	2 (16.7)	0 (0)	0 (0)	0 (0)	2 (9.1)
CG66 ST3252	1 (8.3)	0 (0)	0 (0)	0 (0)	1 (4.5)
CG86 ST86	1 (8.3)	0 (0)	1 (25)	0 (0)	2 (9.1)
CG260 ST260	1 (8.3)	0 (0)	0 (0)	0 (0)	1 (4.5)
CG380 ST380	2 (16.7)	0 (0)	0 (0)	0 (0)	2 (9.1)
CG432 ST432	0 (0)	0 (0)	0 (0)	1 (33.3)	1 (4.5)
CG446 ST446	0 (0)	0 (0)	1 (25)	0 (0)	1 (4.5)
CG827 ST827	0 (0)	0 (0)	0 (0)	1 (33.3)	1 (4.5)
Capsular type					
K1	3 (25)	2 (66.7)	1 (25)	0 (0)	6 (27.3)
K2	7 (58.3)	1 (33.3)	1 (25)	0 (0)	9 (40.9)
Other	2 (16.7)	0 (0)	2 (50)	3 (100)	7 (31.8)

a CG, clonal group; ST, sequence type.

### Virulence genes and MDR convergence.

Virulence and antibiotic resistance elements were always observed in distinct isolates, except for one human K. pneumoniae isolate (KP586) where a convergence of virulence and MDR was observed. It was assigned to ST392, belonging to the successful MDR clonal group CG147. It was community acquired and displayed the aerobactin virulence operon, *bla*_CTX-M-15_, and numerous other resistance genes (*aadA2*, *aac3-IIa*, *bla*_OXA-1_, *bla*_SHV-67_, *bla*_TEM-30_, *catB4*, *dfrA14*, *dfrA32*, *tetA*, *sulI*, and *sulII*).

### Genome-wide association study.

We performed a genome-wide association study (GWAS) on Kp1 isolates in order to identify genes associated with human sources. A genomic region was significantly associated with virulence and host specificity (human). This region includes a cluster of genes located in an integrative conjugative element that mobilizes the *ybt* locus, which encodes the biosynthesis of the siderophore yersiniabactin and its receptor (see Table S8 in the supplemental material). We also found 5 genes significantly associated with human infections encoding proteins with catalytic activity (aconitate hydratase, cytochrome *bo*_3_ ubiquinol oxidase, acetoin:2,6-dichlorophenolindophenol oxidoreductase, and dihydrolipoamide dehydrogenase) and 1 encoding a putative methyltransferase. Two genes were significantly associated with nonhuman isolates, including a gene encoding a core component of a type VI secretion system (T6SS) (T6SS baseplate subunit TssK).

## DISCUSSION

The increasing level of AMR is a major health hazard for humans and animals (19). Tackling AMR transmission requires investigations of the nonclinical reservoirs and their relative contribution to human infections through the so-called One Health approach. WGS combined with phylogenetic analysis is a powerful approach to provide detailed insights into bacterial transmission dynamics. Despite the urgent public health threat represented by the KpSC, only a few studies so far have identified their genomic features using WGS from nonhuman sources and compared these features with those obtained from contemporaneous and colocalized clinical isolates (7–11). We observed a limited overlap of STs between clinical isolates and local nonhuman isolates, consistent with the results of previous studies (here, 12.7% of human STs; range in previous studies, 5 to 15%). In a large survey of an Italian contemporary KpSC collection from a well-defined geographical region, direct transmission from animal or environmental reservoirs represents a small fraction (<1%) of human infections (20). This highlights the difficulty in identifying direct transmission events for a pathogen characterized by its high genetic diversity without a dominance of specific successful lineages. However, we identify evidence of sporadic transmissions between animals and humans in our set of isolates, such as the presence of 3 hospital-acquired human MDR high-risk K. pneumoniae clones (ST17, ST37, and ST45) in pets and 1 successful human hypervirulent K. pneumoniae clone (ST86) in a dog. It should be noted that all but one of the STs (one bovine) were shared only between pets and humans, highlighting the potential risk of companion animals in the transmission of KpSC isolates to humans and vice versa, as previously described (21, 22). The presence of the same isolate (<10 SNPs) assigned to ST45 in a dog and in a human collected 1 month apart highlights the importance of the application of basic hygiene rules for contact with companion animals.

Our results illustrate the efficiency of the genomic approach to distinguish epidemiologically related isolates from unrelated ones within hospitals. Genetically closely related isolates were recovered during the 24-month study across different units of the hospital, suggesting an environmental reservoir and long-term transmission. Most nosocomial isolates belonged to high-risk MDR genetic backgrounds, namely, ST11, ST17, ST45, and ST307. These clones have emerged as important vehicles for the worldwide dissemination of AMR determinants (23, 24), including in the Caribbean islands (16).

Although members of the KpSC can be found in a large variety of ecological niches (25), knowledge of the prevalence and distribution of species and phylogroups belonging to this complex according to the source is limited due to the lack of large-scale systematic sampling efforts. Our findings show that the KpSC is ubiquitous, as shown previously in the environment (25), food (26), and the intestines of mammals (21, 27, 28). To the best of our knowledge, dogs were significantly associated for the first time with a higher risk of KpSC carriage than cats. Consistent with the results of previous studies (8, 20), species and phylogroups were not randomly distributed. In particular, K. pneumoniae *sensu stricto* (Kp1) and Kp3 (*K. variicola* subsp. *variicola*) were clearly separated according to ecological niche: a high prevalence of Kp1 and a low prevalence of Kp3 in humans and domestic and food-producing animals (except for bovines) and the contrary in vegetables and the environment. This may imply ecological barriers that limit the spread of clones and antibiotic resistance genes. However, the distributions of species/phylogroups within the same source were not completely consistent across studies (8, 20), highlighting the need for further work with the inclusion of isolates from wider environmental and animal sources from various geographical areas. For example, we observed a high prevalence of Kp3 among our bovine isolates, probably due to transient flora related to the consumption of raw plants rather than a specific adaptation to colonize their intestine, consistent with the results of a previous study (8) but not with the results of another one (20).

As KpSC isolates were largely recovered in human, animal, and environmental reservoirs in Guadeloupe, we hypothesize that the KpSC could be a major vector for the amplification and spread of antibiotic resistance genes due to its abilities to move between ecological niches, capture and maintain plasmids carrying AMR genes for a long time, and transfer plasmids within KpSC members but also to other important Gram-negative bacteria (6). However, a high level of resistance to antibiotics was rarely found in isolates collected outside the hospital setting but also in isolates of species other than K. pneumoniae, illustrated by the almost exclusive presence of ESBL and carbapenemase genes in human K. pneumoniae isolates, in agreement with the results of previous studies in Guadeloupe (29–31). Nevertheless, further studies using a shotgun metagenomics approach are needed to access the whole resistome in different ecosystems. Despite the risk of the occasional emergence of novel resistance mechanisms in KpSC isolates from environmental sources, our findings strongly support that the nosocomial setting is central to KpSC resistance dissemination, as observed in Europe for carbapenemase-producing K. pneumoniae (32), and that KpSC resistance circulates less frequently between the different compartments.

The *bla*_CTX-M-15_ ESBL gene, first detected in 1999 in India, was the most widely distributed ESBL gene in our set of isolates, in agreement with the worldwide situation. AMR genes and plasmids are often associated with certain K. pneumoniae genetic lineages, as highlighted by the success of K. pneumoniae ST258 being intricately linked with *bla*_KPC_ (33). Further work will be done to characterize the plasmids carrying ESBL genes using Oxford Nanopore technologies in order to study the dissemination of plasmids and their ESBL genes within the hospital setting.

High levels of virulence also tended to be rare in species other than K. pneumoniae. Most of the human HvKp isolates belonged to K. pneumoniae ST23 and more precisely to sublineage CG23-I, which emerged in approximately 1928 following the acquisition of ICEKp10 (encoding yersiniabactin and colibactin) and then spread worldwide (18). The uncommon ST66 genetic background (34) was also identified. A reservoir of HvKp was not found outside humans, but dogs could be an important link in the chain of the transmission of this pathogen (9% of hypervirulent isolates were recovered from this species), as highlighted by the presence of ST86 associated with a dog and a human meningitis case. Aerobactin (*iuc* operon) was present in 90% of our HvKp isolates. It is considered a critical siderophore system of HvKp as it contributes predominantly to hypervirulence in laboratory experiments and mouse models of disease, while the inactivation of other siderophore systems has minimal effects (35, 36). Surprisingly, a high frequency of aerobactin (15%) in K. pneumoniae isolates from pigs was observed, as previously described in Thailand (37) and Germany (11), probably reflecting an adaptation conferred by this siderophore to porcine hosts. Although our isolates were isolated from pigs from different farms and belonged to different STs, they harbored an *iuc3* gene carried by an IncFIB(K) plasmid (for at least two isolates), as observed in Germany (11). These observations suggest that successful IncFIB(K)/*iuc3*-carrying plasmids have spread across wide geographical distances and occur in different K. pneumoniae lineages associated with domestic pigs. The potential risk to animal and human health should also be investigated. Unsurprisingly, a significant association was observed between human infections and the *ybt* locus encoding yersiniabactin, the most common K. pneumoniae high-virulence factor, present in around one-third of clinical isolates (8, 38). The emergence of potentially high-risk MDR and hypervirulent lineages within the hospital setting in Guadeloupe should be monitored, as illustrated by the acquisition of virulence genes in an MDR genetic background (ST392), even if still rarely observed. For the other five genes found to be significantly associated with human infections by GWASs, we did not find any explanation.

Our study represents a large contemporaneous and colocalized sampling and sequencing effort on an island characterized by its small population and area and known to be a hot spot for the spread of health care-associated MDR K. pneumoniae. Despite its insular character, which is expected to promote mainly transmissions with local clones and a restricted contribution from the outside, we found limited evidence for direct transmission between human and nonhuman sources (animals and the environment). In contrast, the nosocomial context seems to be a cornerstone of the dissemination of MDR clones and antibiotic resistance genes.

## MATERIALS AND METHODS

### Fecal samples from healthy food-producing animals and pets.

The study design and methods for selecting healthy food-producing animals were described previously (39). Briefly, between January 2018 and May 2019, fecal samples from pigs and beef cattle were collected randomly just after excretion. Fecal material from 17 hen houses (representing 53,000 poultry) was sampled by walking on litter approximately 100 m around a flock in boot socks. In all, the animals originated from 11 pig farms, 8 beef cattle farms, and 9 poultry farms distributed throughout the island and from the only slaughterhouse in Guadeloupe (34 additional farms) for cattle and pigs. Sixty-four percent of farmers declared antibiotic use during the previous 1 year for curative treatment, with the most commonly used antibiotic being tetracycline (69.0%). No ethics committee approval was necessary as no invasive procedure was conducted on live animals according to French national law for the protection of animals (no. 2013-118), which reproduces European directive 2010/63/EU on the protection of animals used for experimental and other scientific purposes.

For pets, from June to September 2019, a single rectal swab was taken from dogs and cats. The animals were included from the main animal shelter of Guadeloupe and seven veterinary clinics located throughout the territory, among animals sent for preventive health services, vaccination, or medical consultation. With regard to the identification of risk factors for the fecal carriage of ESBL-producing KpSC, pets with clinical signs of diarrhea and/or with antibiotic treatment in the previous month were excluded. The information collected for each animal included age, place of residence, general health, and lifestyle (indoors or wandering free outdoors and close contact with other animals or not). The project was approved by the Committee for Ethics in Animal Experiments of the French West Indies and Guyana (reference no. HC_2020_1).

### Fresh fruits, fresh vegetables, flowering plants, aromatic herbs, and water and soil samples.

Locally produced fresh vegetables, flowering plants, fruits, and aromatic herbs were collected aseptically at four local markets during eight campaigns from January to June 2018. The collected samples spanned producers from 14 municipalities throughout Guadeloupe. Data related to the market and the farm of origin were recorded. When the farm of origin was unknown, multiple vendors in different parts of the market were selected to minimize the likelihood that samples came from the same source.

Raw water samples were collected during the same period at 29 catchment points in 11 municipalities, in partnership with the regional health agency and the hygiene laboratory of the Pasteur Institute of Guadeloupe. These samples corresponded to drinking water before treatment.

From October to December 2019, soil was sampled near rivers and natural ponds: the surface layer (0 to 10 cm) was collected after the removal of plants, pebbles, and conspicuous roots. At each site, two or three samples of soil were taken at least 3 m apart. One sample of water from rivers or natural ponds was collected in proximity. Sampling sites were classified by Q-GIS software into two groups according to their degree of anthropogenic pressure: (i) wilderness with no human presence or countryside with limited human activities, (ii) human-perturbed landscapes with a matrix of agriculture and livestock activities, and (iii) urban and suburban areas with high levels of human activity.

All samples were kept at 2°C to 8°C and processed at the laboratory within 24 h.

### Clinical isolates.

Between January 2018 and December 2019, 279 contemporaneous KpSC isolates were collected from patients admitted to the University Hospital of Guadeloupe, a 900-bed teaching hospital. Isolation was performed as part of the routine activity of the hospital bacteriological diagnostic laboratory. All presumptive HvKp isolates, defined as KpSC isolates associated with community-acquired monomicrobial liver abscess or other monomicrobial invasions of normally sterile sites (e.g., meningitis), were included in the same period. The following metadata were anonymously recorded: date of hospital admission, ward of hospitalization, date and site of sampling, and antimicrobial susceptibility testing results. Isolates were considered to be community acquired if they were recovered by culture from a sample obtained within 48 h after admission in a patient with no risk factors for nosocomial acquisition in the previous year, namely, hospitalization or surgery, the use of an indwelling catheter or a percutaneous device, or frequent exposure to health care facilities for an underlying chronic disorder. All other isolates were considered to be hospital acquired. The study protocol was approved by the ethic committee of the University Hospital of Guadeloupe (reference no. A5_19_12_05_TRAMID).

### K. pneumoniae species complex isolation and antimicrobial susceptibility testing.

Fruits, vegetables, flowering plants, and aromatic herbs after mixing and soil and fecal samples (stool and boot sock samples and endorectal swabs) were inoculated into Luria-Bertani (LB) broth with amoxicillin (10-mg/L final concentration). For water samples, 100 mL of serially diluted samples was filtered through a 0.45-μm membrane filter (Millipore, Guyancourt, France), and the membranes were placed into 9 mL of LB broth with amoxicillin (10-mg/L final concentration). After incubation for 18 h at 37°C, 100 μL of the enrichment culture was plated onto two selective media, Simmons citrate agar (Becton, Dickinson, USA) with 1% inositol (SCAI) medium agar plates for KpSC detection and chromogenic agar (CCA) with ceftriaxone at 4 mg/L (CHROMagar) for the detection of 3GCR KpSC. Presumptive Enterobacteriaceae colonies on selective SCAI medium (large, yellow, glossy colonies) and selective CCA with ceftriaxone (pink colonies), corresponding to oxidase-negative and Gram-negative bacilli, were isolated randomly and identified by matrix-assisted laser desorption ionization–time of flight mass spectrometry (MALDI-TOF MS) on an Axima Performance system (Shimadzu Corp., Japan).

Three colonies were identified randomly for each identical morphology. Susceptibility to amoxicillin (10 μg), amoxicillin-clavulanic acid (20 μg/10 μg), ticarcillin (75 μg), cefotaxime (5 μg), ceftazidime (10 μg), cefepime (30 μg), cefoxitin (30 μg), aztreonam (30 μg), ertapenem (10 μg), gentamicin (15 μg), amikacin (30 μg), trimethoprim-sulfamethoxazole (1.25/23.75 μg), nalidixic acid (30 μg), and ciprofloxacin (5 μg) was tested by the disk diffusion method on Mueller-Hinton agar (Bio-Rad, Marnes-la-Coquette, France), and the production of ESBL was detected by the double-disk synergy test, according to 2020 guidelines of CA-SFM/EUCAST (https://www.sfm-microbiologie.org/2020/10/02/casfm-eucast-v1-2-octobre-2020/). Isolates with a resistant or intermediate phenotype were classified together for analysis. Growth inhibition diameters were measured with the Adagio automated system (Bio-Rad, Marnes-la-Coquette, France). Susceptibility to colistin was determined using a MicroScan Walkaway Plus system (Beckman Coulter, USA). Resistant isolates were defined by an MIC of >4 mg/L. If more than one KpSC isolate with the same antibiotic susceptibility pattern was isolated from the same sample, only the first one was analyzed. An MDR KpSC isolate was defined as an isolate resistant to three or more antimicrobial classes (40).

### DNA extraction, K. pneumoniae species complex identification, and carbapenemase resistance gene screening.

DNA was extracted with a DNA minikit (Qiagen, Germany). A real-time PCR method based on specific sets of primers and probes was used to identify K. pneumoniae isolates to the species and phylogroup levels. The protocols are provided in Text S1 in the supplemental material. All isolates were handled using this method, except for those associated with nosocomial infections, of which about half were randomly selected due to the large number of isolates collected. For isolates with decreased susceptibility to ertapenem, carbapenemase genes were searched for using PCR amplification according to methods described in a previous study (17).

### Genome sequencing and data analysis.

WGS was carried out at the Plateforme de Microbiologie Mutualisée of the Institut Pasteur (Paris, France). Reads were trimmed and filtered with AlienTrimmer software (41), yielding a mean estimated coverage of 86-fold. Genomic assemblies were performed using SPAdes software (42), and the quality of the assembly was evaluated using QUAST software (43). Genomes with a cumulative size of contigs of >6 Mb (expected size of ~5.5 Mb) or a number of contigs of >500 were discarded, as we suspected the presence of multiple clones. The mean *N*_50_ was 197,864 bp.

KpSC phylogrouping, sequence type (ST) assignment, and antibiotic resistance gene detection were performed using Kleborate (44). This tool classified isolates according to the content of resistance genes as follows: 0 for no ESBL and no carbapenemase, 1 for ESBL positive, 2 for carbapenemase positive, and 3 for carbapenemase with colistin resistance (44). Plasmid replicons were identified using the PlasmidFinder database available from ABRicate software, using a minimum coverage and a minimum identity of 90% (45). Kleborate was also used to search for yersiniabactin, colibactin, aerobactin, and salmochelin operons and the presence of *rmpA* and *rmpA2* and to predict capsular types (44). BIGSdb (https://bigsdb.pasteur.fr/klebsiella/) was used to check for the presence of intact *iucABCD-iutA* (aerobactin) and *iroBCDN* (salmochelin) operons. To study the genetic support of *iuc* genes in more detail, the corresponding contigs were predicted to be plasmid or chromosomally associated by combining 2 different software tools (MOB-recon and PlasFlow) with default thresholds (46, 47).

The definitions described previously by Huynh et al. (48) for hypervirulent isolates and successful clones were used. Hypervirulent isolates were defined as isolates harboring at least one of the *rmpA* and *rmpA2* genes and/or at least one complete operon among *iucABCD-iutA* and *iroBCDN*. Successful clones were defined as those belonging to an ST represented at least 10 times in NCBI genomes and mentioned in the title or abstract of at least five publications in NCBI PubMed (“Klebsiella” + “*pneumoniae*” + “ST*xxx*”).

### Pangenome and phylogenetic analyses.

Filtered raw contigs were assigned to the chromosome or plasmid using MOB-suite (47), and chromosomal contigs were then ordered and oriented using RaGOO (49) with a publicly available complete assembled genome available for each Klebsiella species, according to the species assigned previously by Kleborate (44). Genome annotation was performed using Prokka (50), and pangenome analysis was performed using Roary software (51). Pangenome matrix representation was done using the Roary plots python utility (https://github.com/sanger-pathogens/Roary/tree/master/contrib/roary_plots).

Based on the core-genome alignment provided by Roary and after the identification of recombinant regions and the reduction of the alignment using ClonalFrameML (52), a global phylogenetic tree was constructed using RAxML (53) and visualized using iTOL (54). Estimation of the number of single nucleotide polymorphisms (SNPs) between isolates assigned to an identical ST was performed using the PathogenWatch platform (https://pathogen.watch), an online global database for genomic surveillance of KpSC isolates (55). Contigs were uploaded, and collections were created separately by ST, in order to generate SNP difference matrix files on the one hand and Newick files to be visualized as unrooted trees using iTOL on the other hand. Two isolates were considered clonal when the number of SNPs between them was <10. Additional Klebsiella strains obtained from other Caribbean islands (16) were added to the collection. For this purpose, raw FASTQ files were retrieved from the SRA and preliminarily assembled using Unicycler to then be uploaded to PathogenWatch.

The pangenome matrix from Roary consists of gene presence or absence for each genome. It was used as the input for Scoary V1.6.16 (56) in order to search for genes associated with humans or other sources. Due to the bias that might be introduced by the high predominance of Kp1 in human isolates, we focused our pangenome-wide association studies (pan-GWASs) on Kp1 only. Genes returning a Bonferroni-corrected *P* value of ≤0.05 were considered to be significantly present/absent and were further investigated.

### Statistical analyses.

Results were expressed as numbers and frequencies. In bivariate analyses, the χ^2^ test (or Fisher’s exact test when appropriate) was used to compare categorical data between groups. A logistic regression model was performed to identify factors associated with the presence of KpSC isolates and to calculate crude and adjusted odds ratios and their 95% confidence intervals. Factors with a *P* value of <0.20 in the bivariate analysis were retained for the multivariate analysis. For all tests, we considered a *P* value of <0.05 to be significant. Statistical analyses were performed using SPSS (V21; IBM SPSS Statistics, Chicago, IL).

### Data availability.

Reads were deposited in the NCBI SRA public archives under BioProject accession no. PRJNA778230.
