# Supplementary material for: Limited Transmission of Klebsiella pneumoniae among Humans, Animals, and the Environment in a Caribbean Island, Guadeloupe (French West Indies)
Source: Microbiol Spectr. 2022 Sep 12;10(5):e01242-22. doi: 10.1128/spectrum.01242-22 (PMC9603589; doi:10.1128/spectrum.01242-22)
Supplement: Supplemental file 1 — Supplemental material. Download spectrum.01242-22-s0001.pdf, PDF file, 1.5 MB [file spectrum.01242-22-s0001.pdf]

Supplemental Material

Limited transmission of Klebsiella pneumoniae between humans, animals and the environment in a Caribbean island, Guadeloupe (French West Indies)

Alexis Dereeper, Gaëlle Gruel, Matthieu Pot, David Couvin, Elodie Barbier, Sylvaine Bastian, Jean-Christophe Bambou, Moana Gelu-Simeon, Séverine Ferdinand, Stéphanie Guyomard-Rabenirina, Virginie Passet, Frederic Martino, Pascal Piveteau, Yann Reynaud, Carla Rodrigues, Pierre-Marie Roger, Xavier Roy, Antoine Talarmin, Benoit Tressieres, Marc Valette, Sylvain Brisse, Sébastien Breurec

Table S1. Risk factors for *K. pneumoniae* species complex rectal carriage in 185 household and shelter pets from Guadeloupe (French West Indies)

		K. pneumoniae species complex		Univariate analysis		Multivariate analysis
		No	Yes	p-value	OR Brut 95% CI	OR Brut 95% CI
Animal	Cat	40 (40.4)	20 (23.3)	0.013	Réf.	Réf.
	Dog	59 (59.6)	66 (76.7)		2.237 [1.178 - 4.249]	2.231 [1.171 - 4.252]
Sex	Male	44 (44.4)	36 (41.9)	0.723	Réf.	
	Female	55 (55.6)	50 (58.1)		1.111 [0.620 - 1.992]	
Shelter stay	No	90 (90.9)	80 (93.0)	0.599	Réf.	
	Yes	9 (9.1)	6 (7.0)		0.750 [0.256 - 2.200]	
Antibiotic administration ≤ 3 months	No	88 (88.9)	70 (81.4)	0.150	Réf.	
	Yes	11 (11.1)	16 (18.6)		1.829 [0.798 - 4.191]	1.818 [0.782 - 4.225]
Chronic disease	No	93 (93.9)	80 (93.0)	0.801	Réf.	
	Yes	6 (6.1)	6 (7.0)		1.163 [0.361 - 3.747]	
Age ≥ 20 months	No	50 (51.5)	42 (50.6)	0.899	Réf.	
	Yes	47 (48.5)	41 (49.4)		1.039 [0.578 - 1.866]	
Free wandering outdoor	No	93 (93.9)	79 (91.9)	0.581	Réf.	
	Yes	6 (6.1)	7 (8.1)		1.373 [0.443 - 4.255]	
Contact with other animals	No	97 (98.0)	85 (98.8)	1.000	Réf.	
	Yes	2 (2.0)	1 (1.2)		0.571 [0.051 - 6.404]	

Table S2. Resistance to specific antibiotics in 546 *K. pneumoniae* species complex isolates from Guadeloupe (French West Indies) according to the source

	Humans		Pets		Food-producing animals			Environment						Total
	Community	Nosocomial	Dog	Cat	Pig	Bovine	Chicken (litter)	Soil	River / Natural pond	Fruits	Vegetables	Condiments	Water catchment	
	n (%)	n (%)	n (%)	n (%)	n (%)	n (%)	n (%)	n (%)	n (%)	n (%)	n (%)	n (%)	n (%)	n (%)
N	56	223	74	20	30	39	11	14	7	10	42	5	15	546
AMC[a]	6 (10.7)	147 (65.9)	4 (5.4)	2 (10)	3 (10)	2 (5.1)	0 (0)	2 (14.3)	0 (0)	0 (0)	1 (2.4)	0 (0)	0 (0)	167 (30.6)
Cefoxitin	0 (0)	29 (13.0)	0 (0)	0 (0)	0 (0)	0 (0)	0 (0)	0 (0)	0 (0)	0 (0)	0 (0)	0 (0)	0 (0)	29 (5.3)
Cefotaxime	0 (0)	122 (54.7)	0 (0)	1 (5)	0 (0)	0 (0)	0 (0)	0 (0)	0 (0)	0 (0)	0 (0)	0 (0)	0 (0)	123 (22.5)
Ceftazidime	0 (0)	122 (54.7)	0 (0)	1 (5)	0 (0)	0 (0)	0 (0)	0 (0)	0 (0)	0 (0)	0 (0)	0 (0)	0 (0)	123 (22.5)
Cefepim	0 (0)	117 (52.5)	0 (0)	1 (5)	0 (0)	0 (0)	0 (0)	0 (0)	0 (0)	0 (0)	0 (0)	0 (0)	0 (0)	118 (21.6)
Ertapenem	0 (0)	10 (4.5)	0 (0)	0 (0)	0 (0)	0 (0)	0 (0)	0 (0)	0 (0)	0 (0)	0 (0)	0 (0)	0 (0)	10 (1.8)
Nalidixic acid	5 (8.9)	119 (53.4)	0 (0)	1 (5)	0 (0)	0 (0)	0 (0)	0 (0)	0 (0)	0 (0)	0 (0)	0 (0)	0 (0)	125 (22.9)
Ciprofloxacin	4 (7.1)	112 (50.2)	0 (0)	1 (5)	0 (0)	0 (0)	0 (0)	0 (0)	0 (0)	0 (0)	0 (0)	0 (0)	0 (0)	117 (21.4)
Amikacin	0 (0)	20 (9)	0 (0)	1 (5)	0 (0)	0 (0)	0 (0)	0 (0)	0 (0)	0 (0)	0 (0)	0 (0)	0 (0)	21 (3.8)
Gentamicin	0 (0)	95 (42.6)	0 (0)	0 (0)	0 (0)	0 (0)	0 (0)	0 (0)	0 (0)	0 (0)	0 (0)	0 (0)	0 (0)	95 (17.4)
SXT[b]	5 (8.9)	111 (49.8)	1 (1.4)	1 (5)	3 (10)	0 (0)	2 (18.2)	0 (0)	0 (0)	0 (0)	0 (0)	0 (0)	0 (0)	123 (22.5)
ESBL[c]	0 (0)	116 (52.0)	0 (0)	1 (5)	0 (0)	0 (0)	0 (0)	0 (0)	0 (0)	0 (0)	0 (0)	0 (0)	0 (0)	117 (21.4)
CoP[d]	0 (0)	12 (5.4)	0 (0)	0 (0)	0 (0)	0 (0)	0 (0)	0 (0)	0 (0)	0 (0)	0 (0)	0 (0)	0 (0)	12 (2.2)
Carbapenemase	0 (0)	3 (1.3)	0 (0)	0 (0)	0 (0)	0 (0)	0 (0)	0 (0)	0 (0)	0 (0)	0 (0)	0 (0)	0 (0)	3 (0.5)
Wild-type phenotype	47 (82.1)	71 (31.8)	69 (93.2)	18 (90)	25 (83.3)	37 (94.9)	9 (81.8)	12 (85.7)	7 (100)	10 (100)	41 (97.6)	5 (100)	15 (100)	365 (66.8)
MDR[e]	0 (0)	112 (50.2)	0 (0)	1 (5)	0 (0)	0 (0)	0 (0)	0 (0)	0 (0)	0 (0)	0 (0)	0 (0)	0 (0)	113 (20.7)

[a]Amoxicillin/clavulanic acid, [b]Trimethoprim/sulfamethoxazole, [c]Extended-spectrum β-lactamase, [d]Plasmid-mediated *ampC* gene, [e]Multidrug resistant

Table S3. Species and phylogroups distribution of 433 *K. pneumoniae* species complex isolates from Guadeloupe (French West Indies) according to the source by RT-PCR. Phylogroup typing was performed for all the isolates, except for those recovered in the hospital setting (around 50% were tested)

	Humans		Pets		Food-producing animals			Environment						Total
	Community	Nosocomial	Dog	Cat	Pig	Bovine	Chicken (litter)	Soil	River / Natural pond	Fruits	Vegetables	Condiments	Water catchment	
	n (%)	n (%)	n (%)	n (%)	n (%)	n (%)	n (%)	n (%)	n (%)	n (%)	n (%)	n (%)	n (%)	n (%)
N	56	110	74	20	30	39	11	14	7	10	42	5	15	433
Kp1	46 (82.1)	99 (90.0)	55 (74.3)	17 (85)	13 (43.3)	2 (5.1)	6 (54.5)	1 (7.1)	2 (28.6)	1 (10)	1 (2.4)	0 (0)	2 (13.3)	245 (56.6)
Kp2	1 (1.8)	2 (1.8)	5 (6.8)	1 (5)	11 (36.7)	13 (33.3)	3 (27.3)	2 (14.3)	1 (14.3)	3 (30)	18 (42.9)	1 (20)	1 (6.7)	62 (14.3)
Kp3/Kp5	2 (3.6)	6 (5.5)	5 (6.8)	1 (5)	4 (13.3)	16 (41)	2 (18.2)	4 (28.6)	4 (57.1)	4 (40)	19 (45.2)	3 (60)	11 (73.3)	81 (18.7)
Kp4	7 (12.5)	3 (2.7)	9 (12.2)	1 (5)	2 (6.7)	8 (20.5)	0 (0)	7 (50)	0 (0)	2 (20)	4 (9.5)	1 (20)	1 (6.7)	45 (10.4)
p	0.09		0.914		<0.001			0.016						

Table S5. Acquired antimicrobial resistance genes in 331 *K. pneumoniae* species complex isolates from Guadeloupe (French West Indies) according to the source

	Humans		Pets		Food-producing animals			Environment						Total
	Community	Nosocomial	Dog	Cat	Pig	Bovine	Chicken (litter)	Soil	River/Natural pond	Fruits	Vegetables	Flowering plants	Water catchment	
	n (%)	n (%)	n (%)	n (%)	n (%)	n (%)	n (%)	n (%)	n (%)	n (%)	n (%)	n (%)	n (%)	n (%)
N	56	97	45	11	20	31	9	12	7	1	28	8	6	331
arr3	2 (3.6)	8 (8.2)	0 (0)	0 (0)	0 (0)	0 (0)	0 (0)	0 (0)	0 (0)	0 (0)	0 (0)	0 (0)	0 (0)	10 (3)
aadA-like	4 (7.1)	18 (18.6)	0 (0)	0 (0)	1 (5)	0 (0)	1 (11.1)	0 (0)	0 (0)	0 (0)	0 (0)	0 (0)	0 (0)	24 (7.3)
aac(3')-IId	0 (0)	2 (2.1)	0 (0)	0 (0)	0 (0)	0 (0)	0 (0)	0 (0)	0 (0)	0 (0)	0 (0)	0 (0)	0 (0)	2 (0.6)
aac(3)-IIa	0 (0)	45 (46.4)	0 (0)	0 (0)	0 (0)	0 (0)	0 (0)	0 (0)	0 (0)	0 (0)	0 (0)	0 (0)	0 (0)	45 (13.6)
aac(6')-Ic	0 (0)	0 (0)	0 (0)	0 (0)	0 (0)	0 (0)	0 (0)	0 (0)	1 (14.3)	0 (0)	0 (0)	0 (0)	0 (0)	1 (0.3)
aac(6')-Ib	0 (0)	2 (2.1)	0 (0)	0 (0)	0 (0)	0 (0)	0 (0)	0 (0)	0 (0)	0 (0)	0 (0)	0 (0)	0 (0)	2 (0.6)
aph(3')-Ia	0 (0)	2 (2.1)	0 (0)	0 (0)	0 (0)	0 (0)	0 (0)	0 (0)	0 (0)	0 (0)	0 (0)	0 (0)	0 (0)	2 (0.6)
catA-like	1 (1.8)	7 (7.2)	0 (0)	0 (0)	0 (0)	0 (0)	0 (0)	0 (0)	0 (0)	0 (0)	0 (0)	0 (0)	0 (0)	8 (2.4)
catB-like	1 (1.8)	20 (20.6)	0 (0)	0 (0)	0 (0)	0 (0)	0 (0)	0 (0)	0 (0)	0 (0)	0 (0)	0 (0)	0 (0)	21 (6.3)
cmlA5	0 (0)	1 (1)	0 (0)	0 (0)	0 (0)	0 (0)	0 (0)	0 (0)	0 (0)	0 (0)	0 (0)	0 (0)	0 (0)	1 (0.3)
dfrA-like	6 (10.7)	61 (62.9)	0 (0)	1 (9.1)	1 (5)	0 (0)	1 (11.1)	0 (0)	0 (0)	0 (0)	0 (0)	0 (0)	0 (0)	70 (21.1)
mpHA	0 (0)	4 (4.1)	0 (0)	0 (0)	0 (0)	0 (0)	0 (0)	0 (0)	0 (0)	0 (0)	0 (0)	0 (0)	0 (0)	4 (1.2)
mgrB	0 (0)	1 (1)	0 (0)	0 (0)	0 (0)	0 (0)	0 (0)	0 (0)	0 (0)	0 (0)	0 (0)	0 (0)	0 (0)	1 (0.3)
Mutations QRDR[1]	4 (7.1)	42 (43.3)	0 (0)	0 (0)	0 (0)	0 (0)	0 (0)	0 (0)	0 (0)	0 (0)	0 (0)	0 (0)	0 (0)	46 (13.9)
qnrB	2 (3.6)	47 (48.5)	0 (0)	1 (9.1)	0 (0)	0 (0)	1 (11.1)	0 (0)	0 (0)	0 (0)	0 (0)	0 (0)	0 (0)	51 (15.4)
strA	2 (3.6)	40 (41.2)	1 (2.2)	1 (9.1)	6 (30)	0 (0)	0 (0)	0 (0)	0 (0)	0 (0)	0 (0)	0 (0)	0 (0)	50 (15.1)
strB	2 (3.6)	41 (42.3)	1 (2.2)	1 (9.1)	6 (30)	0 (0)	0 (0)	0 (0)	0 (0)	0 (0)	0 (0)	0 (0)	0 (0)	51 (15.4)
sul1	4 (7.1)	19 (19.6)	0 (0)	0 (0)	1 (5)	0 (0)	2 (22.2)	0 (0)	0 (0)	0 (0)	0 (0)	0 (0)	0 (0)	26 (7.9)
sul2	1 (1.8)	42 (43.3)	0 (0)	1 (9.1)	0 (0)	0 (0)	0 (0)	0 (0)	0 (0)	0 (0)	0 (0)	1 (12.5)	0 (0)	45 (13.6)
tetA	0 (0)	38 (39.2)	0 (0)	1 (9.1)	1 (5)	0 (0)	2 (22.2)	0 (0)	0 (0)	0 (0)	0 (0)	0 (0)	0 (0)	42 (12.7)
tetB	0 (0)	0 (0)	0 (0)	0 (0)	3 (15)	0 (0)	0 (0)	0 (0)	0 (0)	0 (0)	0 (0)	0 (0)	0 (0)	3 (0.9)

tetC	0 (0)	0 (0)	0 (0)	0 (0)	3 (15)	0 (0)	0 (0)	0 (0)	0 (0)	0 (0)	0 (0)	0 (0)	0 (0)	3 (0.9)
tetD	4 (7.1)	13 (13.4)	0 (0)	1 (9.1)	1 (5)	0 (0)	0 (0)	0 (0)	0 (0)	0 (0)	0 (0)	0 (0)	0 (0)	19 (5.7)
bla$_{\text{OXA-1}}$	1 (1.8)	47 (48.5)	0 (0)	0 (0)	0 (0)	0 (0)	0 (0)	0 (0)	0 (0)	0 (0)	0 (0)	0 (0)	0 (0)	48 (14.5)
bla$_{\text{OXA-9}}$	0 (0)	2 (2.1)	0 (0)	0 (0)	0 (0)	0 (0)	0 (0)	0 (0)	0 (0)	0 (0)	0 (0)	0 (0)	0 (0)	2 (0.6)
bla$_{\text{DHA-1}}$	0 (0)	3 (3.1)	0 (0)	0 (0)	0 (0)	0 (0)	1 (11.1)	0 (0)	0 (0)	0 (0)	0 (0)	0 (0)	0 (0)	4 (1.2)
bla$_{\text{KPC-2}}$	0 (0)	2 (2.1)	0 (0)	0 (0)	0 (0)	0 (0)	0 (0)	0 (0)	0 (0)	0 (0)	0 (0)	0 (0)	0 (0)	2 (0.6)
bla$_{\text{CTX-M-15}}$	1 (1.8)	57 (58.8)	0 (0)	1 (9.1)	0 (0)	0 (0)	0 (0)	0 (0)	0 (0)	0 (0)	0 (0)	0 (0)	0 (0)	59 (17.8)
bla$_{\text{SHV12}}$	0 (0)	3 (3.1)	0 (0)	0 (0)	0 (0)	0 (0)	0 (0)	0 (0)	0 (0)	0 (0)	0 (0)	0 (0)	0 (0)	3 (0.9)
No ESBL *bla*$_{\text{TEM}}$	0 (0)	56 (57.7)	0 (0)	1 (9.1)	0 (0)	0 (0)	0 (0)	0 (0)	0 (0)	0 (0)	0 (0)	0 (0)	0 (0)	57 (17.2)

[1]Quinolone resistance-determining regions in the *gyrA* and *parC* genes

Table S6. Plasmid incompatibility group from 331 *K. pneumoniae* species complex isolates from Guadeloupe (French West Indies) according to the source

	Humans		Pets		Food-producing animals			Environment						Total
	Community	Nosocomial	Dog	Cat	Pig	Bovine	Chicken (litter)	Soil	River/Natural pond	Fruits	Vegetables	Flowering plants	Water catchment	
	n (%)	n (%)	n (%)	n (%)	n (%)	n (%)	n (%)	n (%)	n (%)	n (%)	n (%)	n (%)	n (%)	n (%)
N	56	97	45	11	20	31	9	12	7	1	28	8	6	331
ColRNAI	7 (12.5)	25 (25.8)	2 (4.4)	1 (9.1)	2 (10)	0 (0)	0 (0)	0 (0)	0 (0)	0 (0)	0 (0)	0 (0)	0 (0)	37 (11.2)
ColpVC	0 (0)	2 (2.1)	0 (0)	0 (0)	0 (0)	0 (0)	0 (0)	0 (0)	0 (0)	0 (0)	0 (0)	0 (0)	0 (0)	2 (0.6)
IncFIA(HI1)	7 (12.5)	7 (7.2)	8 (17.8)	1 (9.1)	3 (15)	2 (6.5)	2 (22.2)	0 (0)	0 (0)	0 (0)	3 (10.7)	2 (25)	0 (0)	35 (10.6)
IncFIB(AP001918)	1 (1.8)	1 (1)	0 (0)	0 (0)	0 (0)	0 (0)	0 (0)	0 (0)	0 (0)	0 (0)	0 (0)	0 (0)	0 (0)	2 (0.6)
IncFIB(K)	44 (78.6)	65 (67)	27 (60)	8 (72.7)	15 (75)	4 (12.9)	1 (11.1)	5 (41.7)	1 (14.3)	0 (0)	10 (35.7)	2 (25)	0 (0)	182 (55.0)
IncFIB(Mar)	0 (0)	8 (8.2)	0 (0)	0 (0)	1 (5)	1 (3.2)	0 (0)	0 (0)	0 (0)	0 (0)	0 (0)	0 (0)	0 (0)	10 (3.0)
IncFIB(pKPHS1)	4 (7.1)	3 (3.1)	1 (2.2)	0 (0)	3 (15)	2 (6.5)	0 (0)	2 (16.7)	1 (14.3)	0 (0)	0 (0)	2 (25)	1 (16.7)	19 (5.7)
Aph(3')-Ia	0 (0)	2 (2.1)	0 (0)	0 (0)	0 (0)	0 (0)	0 (0)	0 (0)	0 (0)	0 (0)	0 (0)	0 (0)	0 (0)	2 (0.6)
IncFIB(pQil)	1 (1.8)	3 (3.1)	1 (2.2)	0 (0)	0 (0)	0 (0)	0 (0)	0 (0)	0 (0)	0 (0)	0 (0)	0 (0)	0 (0)	5 (1.5)
IncFII(29)	1 (1.8)	1 (1)	0 (0)	0 (0)	0 (0)	0 (0)	0 (0)	0 (0)	0 (0)	0 (0)	0 (0)	0 (0)	0 (0)	2 (0.6)
IncFII(p14)	0 (0)	1 (1)	0 (0)	0 (0)	0 (0)	0 (0)	0 (0)	0 (0)	0 (0)	0 (0)	0 (0)	0 (0)	0 (0)	1 (0.3)
IncFII(pCoo)	0 (0)	0 (0)	0 (0)	1 (9.1)	0 (0)	0 (0)	0 (0)	0 (0)	0 (0)	0 (0)	0 (0)	0 (0)	0 (0)	1 (0.3)
IncFII(pKPX1)	0 (0)	1 (1)	0 (0)	0 (0)	0 (0)	0 (0)	0 (0)	0 (0)	0 (0)	0 (0)	0 (0)	0 (0)	0 (0)	1 (0.3)
IncFII(pRSB107)	0 (0)	0 (0)	0 (0)	0 (0)	1 (5)	0 (0)	0 (0)	0 (0)	0 (0)	0 (0)	0 (0)	0 (0)	0 (0)	1 (0.3)
IncFII	10 (17.9)	10 (10.3)	2 (4.4)	1 (9.1)	3 (15)	1 (3.2)	1 (11.1)	1 (8.3)	0 (0)	0 (0)	4 (14.3)	2 (25)	0 (0)	35 (10.6)
IncL/M(pMU407)	0 (0)	0 (0)	0 (0)	2 (18.2)	0 (0)	0 (0)	1 (11.1)	0 (0)	0 (0)	0 (0)	0 (0)	0 (0)	0 (0)	3 (0.9)
IncN	2 (3.6)	0 (0)	0 (0)	0 (0)	2 (10)	0 (0)	0 (0)	0 (0)	0 (0)	0 (0)	0 (0)	0 (0)	0 (0)	4 (1.2)
IncR	12 (21.4)	29 (29.9)	5 (11.1)	2 (18.2)	3 (15)	0 (0)	1 (11.1)	0 (0)	0 (0)	0 (0)	0 (0)	1 (12.5)	0 (0)	53 (16.0)
IncX1	0 (0)	0 (0)	0 (0)	0 (0)	1 (5)	0 (0)	0 (0)	0 (0)	0 (0)	0 (0)	0 (0)	0 (0)	0 (0)	1 (0.3)
IncX3	0 (0)	2 (2.1)	0 (0)	0 (0)	0 (0)	0 (0)	0 (0)	0 (0)	0 (0)	0 (0)	0 (0)	0 (0)	0 (0)	2 (0.6)
IncX4	0 (0)	0 (0)	0 (0)	1 (9.1)	0 (0)	0 (0)	0 (0)	0 (0)	0 (0)	0 (0)	0 (0)	0 (0)	0 (0)	1 (0.3)

Table S7. Virulence genes in 331 *K. pneumoniae* species complex isolates from Guadeloupe (French West Indies) according to the source

	Human		Pets		Food-producing animals			Environment						Total
	Community	Nosocomial	Dog	Cat	Pig	Bovine	Chicken (litter)	Soil	River/Natural pond	Fruits	Vegetables	Flowering plants	Water catchment	
	n (%)	n (%)	n (%)	n (%)	n (%)	n (%)	n (%)	n (%)	n (%)	n (%)	n (%)	n (%)	n (%)	n (%)
N	56	97	45	11	20	31	9	12	7	1	28	8	6	331
Yersiniabactin (*ybt*)	26 (46.4)	41 (42.3)	7 (15.6)	3 (27.3)	2 (10)	0 (0)	0 (0)	0 (0)	0 (0)	0 (0)	1 (3.6)	0 (0)	0 (0)	80 (24.2)
ybt 1 ; ICEKp10	4 (7.1)	1 (1)	0 (0)	0 (0)	0 (0)	0 (0)	0 (0)	0 (0)	0 (0)	0 (0)	0 (0)	0 (0)	0 (0)	5 (1.5)
ybt 12; ICEKp10	3 (5.4)	0 (0)	0 (0)	0 (0)	0 (0)	0 (0)	0 (0)	0 (0)	0 (0)	0 (0)	0 (0)	0 (0)	0 (0)	3 (0.9)
ybt 10; ICEKp4	7 (12.5)	32 (33)	1 (2.2)	1 (9.1)	0 (0)	0 (0)	0 (0)	0 (0)	0 (0)	0 (0)	0 (0)	0 (0)	0 (0)	41 (12.4)
ybt 13; ICEKp2	0 (0)	1 (1)	0 (0)	0 (0)	0 (0)	0 (0)	0 (0)	0 (0)	0 (0)	0 (0)	0 (0)	0 (0)	0 (0)	1 (0.3)
ybt 14; ICEKp5	1 (1.8)	1 (1)	0 (0)	1 (9.1)	0 (0)	0 (0)	0 (0)	0 (0)	0 (0)	0 (0)	0 (0)	0 (0)	0 (0)	3 (0.9)
ybt 15; ICEKp11	1 (1.8)	0 (0)	0 (0)	0 (0)	0 (0)	0 (0)	0 (0)	0 (0)	0 (0)	0 (0)	0 (0)	0 (0)	0 (0)	1 (0.3)
ybt 16; ICEKp12	1 (1.8)	0 (0)	0 (0)	0 (0)	0 (0)	0 (0)	0 (0)	0 (0)	0 (0)	0 (0)	0 (0)	0 (0)	0 (0)	1 (0.3)
ybt 17; ICEKp10	4 (7.1)	1 (1)	0 (0)	0 (0)	0 (0)	0 (0)	0 (0)	0 (0)	0 (0)	0 (0)	0 (0)	0 (0)	0 (0)	5 (1.5)
ybt 2; ICEKp1	0 (0)	0 (0)	3 (6.7)	0 (0)	0 (0)	0 (0)	0 (0)	0 (0)	0 (0)	0 (0)	0 (0)	0 (0)	0 (0)	3 (0.9)
ybt 4; plasmid	2 (3.6)	2 (2.1)	2 (4.4)	1 (9.1)	0 (0)	0 (0)	0 (0)	0 (0)	0 (0)	0 (0)	1 (3.6)	0 (0)	0 (0)	8 (2.4)
ybt 5; ICEKp6	0 (0)	1 (1)	0 (0)	0 (0)	0 (0)	0 (0)	0 (0)	0 (0)	0 (0)	0 (0)	0 (0)	0 (0)	0 (0)	1 (0.3)
ybt 6 ; ICEKp5	1 (1.8)	0 (0)	0 (0)	0 (0)	0 (0)	0 (0)	0 (0)	0 (0)	0 (0)	0 (0)	0 (0)	0 (0)	0 (0)	1 (0.3)
ybt 9; ICEKp3	1 (1.8)	2 (2.1)	0 (0)	0 (0)	0 (0)	0 (0)	0 (0)	0 (0)	0 (0)	0 (0)	0 (0)	0 (0)	0 (0)	3 (0.9)
ybt unknown	1 (1.8)	0 (0)	1 (2.2)	0 (0)	2 (10)	0 (0)	0 (0)	0 (0)	0 (0)	0 (0)	0 (0)	0 (0)	0 (0)	4 (1.2)
colibactin (*clb*)	12 (21.4)	3 (3.1)	0 (0)	0 (0)	0 (0)	0 (0)	0 (0)	0 (0)	0 (0)	0 (0)	0 (0)	0 (0)	0 (0)	15 (4.5)
clb 1	4 (7.1)	0 (0)	0 (0)	0 (0)	0 (0)	0 (0)	0 (0)	0 (0)	0 (0)	0 (0)	0 (0)	0 (0)	0 (0)	4 (1.2)
clb 2	4 (7.1)	2 (2.1)	0 (0)	0 (0)	0 (0)	0 (0)	0 (0)	0 (0)	0 (0)	0 (0)	0 (0)	0 (0)	0 (0)	6 (1.8)
clb 3	4 (7.1)	1 (1)	0 (0)	0 (0)	0 (0)	0 (0)	0 (0)	0 (0)	0 (0)	0 (0)	0 (0)	0 (0)	0 (0)	5 (1.5)
aerobactin (*iuc*)	12 (21.4)	3 (3.1)	2 (4.4)	0 (0)	3 (15)	0 (0)	0 (0)	0 (0)	0 (0)	0 (0)	0 (0)	0 (0)	0 (0)	20 (6)
Iuc 1	7 (12.5)	2 (2.1)	1 (2.2)	0 (0)	0 (0)	0 (0)	0 (0)	0 (0)	0 (0)	0 (0)	0 (0)	0 (0)	0 (0)	10 (3)
Iuc 2	5 (8.9)	1 (1)	0 (0)	0 (0)	0 (0)	0 (0)	0 (0)	0 (0)	0 (0)	0 (0)	0 (0)	0 (0)	0 (0)	6 (1.8)

	Human		Pets		Food-producing animals			Environment						Total
	Community	Nosocomial	Dog	Cat	Pig	Bovine	Chicken (litter)	Soil	River/Natural pond	Fruits	Vegetables	Flowering plants	Water catchment	
Iuc 3	0 (0)	0 (0)	1 (2.2)	0 (0)	3 (15)	0 (0)	0 (0)	0 (0)	0 (0)	0 (0)	0 (0)	0 (0)	0 (0)	4 (1.2)
salmochelin (*iro*)	12 (21.4)	3 (3.1)	4 (8.9)	0 (0)	0 (0)	0 (0)	0 (0)	0 (0)	0 (0)	0 (0)	0 (0)	0 (0)	0 (0)	19 (5.7)
iro 1	7 (12.5)	2 (2.1)	1 (2.2)	0 (0)	0 (0)	0 (0)	0 (0)	0 (0)	0 (0)	0 (0)	0 (0)	0 (0)	0 (0)	10 (3)
iro 2	5 (8.9)	1 (1)	0 (0)	0 (0)	0 (0)	0 (0)	0 (0)	0 (0)	0 (0)	0 (0)	0 (0)	0 (0)	0 (0)	6 (1.8)
iro 3	0 (0)	0 (0)	3 (6.7)	0 (0)	0 (0)	0 (0)	0 (0)	0 (0)	0 (0)	0 (0)	0 (0)	0 (0)	0 (0)	3 (0.9)
rmpA	5 (8.9)	2 (2.1)	2 (4.4)	0 (0)	0 (0)	0 (0)	0 (0)	0 (0)	0 (0)	0 (0)	0 (0)	0 (0)	0 (0)	9 (2.7)
rmpA2	0 (0)	0 (0)	1 (2.2)	0 (0)	0 (0)	0 (0)	0 (0)	0 (0)	0 (0)	0 (0)	0 (0)	0 (0)	0 (0)	1 (0.3)
Allantoinase	4 (7.1)	4 (4.1)	1 (2.2)	0 (0)	0 (0)	2 (6.5)	0 (0)	3 (25)	0 (0)	0 (0)	0 (0)	0 (0)	0 (0)	14 (4.2)
K1	3 (5.4)	2 (2.1)	1 (2.2)	0 (0)	0 (0)	0 (0)	0 (0)	0 (0)	0 (0)	0 (0)	1 (3.6)	0 (0)	0 (0)	7 (2.1)
K2	8 (14.3)	2 (2.1)	1 (2.2)	0 (0)	0 (0)	0 (0)	0 (0)	0 (0)	0 (0)	0 (0)	0 (0)	0 (0)	0 (0)	11 (3.3)

Supplementary Materials and Methods 1

Detection of individual *Klebsiella pneumoniae* phylogroups by real-time PCR

Elodie Barbier[a], Carla Rodrigues[b], Sylvain Brisse[b], Pascal Piveteau[c]
[a] UMR AgroEcologie, INRAE, Bourgogne Franche-Comté University, Dijon, France
[b] Institut Pasteur, University Paris Cité, Biodiversity and Epidemiology of Bacterial Pathogens, Paris, France
[c] UR OPAALE, INRAE, Rennes, France

***In silico* identification of candidate genes for phylogroup identification**

A collection of 66 reference genomes (Table 1) from Institut Pasteur (n=52) and public databases (n=14), including representatives of Kp1 to Kp6 phylogroups, was used to construct a pangenome with Roary v3.11 (1) using a blastP identity cut-off of 80%, and with core genes being defined as those present in more than 90% of the isolates. From the pangenome, we have selected genes exclusive of each phylogroup (i.e. unique genes for Kp1, Kp2, etc), with a total of 96 candidate target genes being obtained (n=8 for Kp1; n=5 for Kp2, n=32 for Kp3 and Kp5, n=9 for Kp4, n=42 for Kp6). No exclusive genes were found to Kp3, so we selected genes targeting Kp3 and Kp5 simultaneously. Note that Kp7 (*K. africana*) was not discovered at the time of these analyses. The specificity of each candidate was checked using BLAST with customized parameters against: (i) all available *Klebsiella* spp. genomes; and (ii) all available genomes excluding *Klebsiella* spp (NCBI GenBank; last accessed: February 2018). After filtering based on G+C content (retention criteria: between 50% and 60%), 19 candidates target genes were selected.

Distribution of the selected target genes in the *K. pneumoniae* species complex

The phylogenetic distribution of candidate target genes was assessed using a previous genomic collection of 1,001 genomes from NCBI GenBank (February 2018) and Institut Pasteur's internal collection, and which included *Klebsiella* spp. and closely related species (*Raoultella* spp.). The full description of this final collection is described elsewhere (2) Nucleotide BLAST (BLASTN) was used to detect the presence of the target sequences in the 1,001 genomes collection, using 80% nucleotide identity and 80% length coverage as cutoffs. A phylogenetic tree was constructed (3)

and the output visualized using iTOL (4). After visual inspection of the distribution of gene presence across the phylogenetic tree leaves, five candidate genes were selected (Fig. 1).

Table 1. Reference genomes used to obtain the pangenome and to search for specific target genes for each *K. pneumoniae* phylogroup.

Taxonomic Designation (PhG)	Strain Name	Strain bank (SB) ID[a]	Isolation Year	Origin	Source	Country	ST	Accession no.
***K. pneumoniae* (Kp1)**	04A025	**SB20**	1997	Human	Blood	Paris	**15**	ERS2786696
	MGH 78578	**SB107**	1994	Human	Blood	n.a.	**38**	GCF_000016315.1
	5-2	**SB617**	2000	Water	Natuurgebied canal	The Netherlands	**55**	ERS2786695
	-	**SB615**	2000	Water	n.a.	The Netherlands	**62**	GCF_020525945.1
	SB4-2	**SB1067**	2002	Human	Feces	The Netherlands	**17**	ERS2786693
	-	**SB1139**	2002	Human	Feces	The Netherlands	**37**	ERS2786694
	-	**SB1170**	2002	Human	Feces	The Netherlands	**45**	GCF_020525645.1
	cur15505	**SB2390**	2002	Human	Urinary tract	n.a.	**14**	GCF_000689275.1
	03-9138	**SB3332**	2003	Human	Anal abscess	Martinique	**65**	n.a.
	Kp 52.145	**SB3341**	1935	Human	n.a.	Indonesia	**66**	GCF_000968155.1
	NTUH-K2044	**SB3928**	1996	Human	Blood	Taiwan	**23**	GCF_000009885.1
	CG43	**SB4454**	n.a.	Human	Liver abscess	Taiwan	**86**	GCF_000474015.1
	BJ1-GA	**SB4496**	2011	Human	Liver abscess	France	**380**	ERS2786698
	T69	**SB4536**	2010	Human	Liver abscess	France	**375**	GCF_020525865.1
	Kp13	**SB4938**	2009	Human	Blood	Brazil	**442**	GCF_000512165.1
	NJST258_1	**SB4975**	2010	Human	Urinary tract	USA	**258**	GCF_000598005.1
***K. quasipneumoniae* subsp *quasipneumoniae* (Kp2)**	01A030T	**SB11**	1997	Human	Blood	Austria	**1528**	GCF_000751755
	18A069	**SB59**	1997	Human	Blood	Spain	**1118**	ERS2786701
	Kleb Ali 0320584	**SB98**	n.a.	n.a.	n.a.	n.a.	**622**	ERS2786702
	-	**SB1124**	2002	Water	n.a.	The Netherlands	**2274**	ERS2786699
	U41	**SB2110**	1990	Environment	n.a.	Germany	**526**	ERS2786700
	1214	**SB5487**	2016	Human	Feces	Madagascar	**2507**	ERS3557014
	1281	**SB5543**	2016	Human	Feces	Madagascar	**526**	ERS3557044

	1284	**SB5545**	2016	Human	Feces	Madagascar	**2604**	ERS3557045
	1385	**SB5617**	2016	Human	Feces	Madagascar	**526**	ERS3557081
	1392	**SB5621**	2016	Human	Feces	Madagascar	**526**	ERS3556845
***K. quasipneumoniae* subsp *similiipneumoniae* (Kp4)**	07A044T	**SB30**	1997	Human	Blood	Germany	**1215**	GCF_000613225.1
	09A323	**SB164**	1997	Human	Blood	Greece	**414**	ERS2786705
	12A476	**SB203**	1998	Human	Blood	Netherlands	**384**	ERS2786706
	CIP110288	**SB4697**	n.a.	Environment	Farmland soil	China	**2275**	ERS2786707
	857	**SB5420**	2016	Human	Feces	Madagascar	**841**	ERS3556979
	1196	**SB5477**	2015	Human	Feces	Madagascar	**2503**	ERS3556801
	1268	**SB5533**	2016	Human	Feces	Madagascar	**2562**	ERS3556843
	1367	**SB5607**	2016	Human	Feces	Madagascar	**1584**	ERS3557075
	ATCC 700603	-	1994	Human	n.a.	USA	**489**	GCF_003181175.1
	HKUOPA4	-	2013	Giant panda	Feces	China	**3870**	GCF_002240295.1
***K. variicola* subsp. *variicola* (Kp3)**	01A065	**SB1**	1997	Human	Blood	Austria	**2273**	ERS2786703
	F2R9T	**SB48**	n.a.	Food	Banana	Mexico	**2263**	ERS2786704
	Kp342	**SB579**	n.a.	Plant	Maize endophytic diazotroph	USA	**146**	GCF_000019565.1
	At22	**SB4767**	n.a.	Environment	Atta cephalotes fungus garden	n.a.	**1220**	GCF_000025465.1
	806	**SB5379**	2015	Human	Feces	Madagascar	**906**	ERS3556874
	869	**SB5430**	2016	Human	Feces	Madagascar	**919**	ERS3556984
	1232	**SB5505**	2016	Human	Feces	Madagascar	**2553**	ERS3557026
	1355	**SB5600**	2016	Human	Feces	Madagascar	**2594**	ERS3557069
	DX120E	-	2008	Environment	Sugarcane root	China	**3926**	GCF_000812205.2
	Gj1	-	2014	Water	River	South Korea	**363**	GCF_001989495.1
***K. variicola* subsp. *tropica* (Kp5)**	CDC 4241-71	**SB94**	n.a.	Environment	n.a.	n.a.	**1216**	ERS2786710
	814	**SB5387**	2015	Human	Feces	Madagascar	**2466**	ERS2787526

	885	**SB5439**	2016	Human	Feces	Madagascar	**2486**	ERS2787527
	1266T	**SB5531**	2016	Human	Feces	Madagascar	**2561**	ERS2787528
	1283	**SB5544**	2016	Human	Feces	Madagascar	**2605**	ERS2787529
	1375	**SB5610**	2016	Human	Feces	Madagascar	**2600**	ERS2787530
	01-436MBV	-	2014	Human	Vaginal swab	Madagascar	**3306**	n.a.
	02-174MBV	-	2013	Human	Vaginal swab	Madagascar	**3316**	n.a.
	02-182MBV	-	2013	Human	Vaginal swab	Madagascar	**3316**	n.a.
	02-846MBV	-	2015	Human	Vaginal swab	Madagascar	**3323**	n.a.
**'*K. quasivariicola*'**	08A119	**SB33**	1997	Human	Blood	Germany	**1214**	ERS2786709
**(Kp6)**	10982	**SB6071**	2005	Human	Peri-rectal	USA	**1155**	GCA_000523395.1
	01-467-2ECBU	**SB6094**	2015	Human	Urinary tract	Madagascar	**2830**	n.a.
	KPN1705T	**SB6096**	2014	Human	Wound	USA	**209**	GCA_002269255.1
	01-467-1ECBU	-	2014	Human	Urinary tract	Madagascar	**2830**	n.a.
	PO552	-	2009	Human	Blood	Nigeria	**3897**	GCA_002186515.2
	VRCO0126	-	2014	Environment	Wastewater	United Kingdom	**4706**	ERR972056
	VRCO0168	-	2015	Environment	n.a.	United Kingdom	**4708**	ERR1796023
	SAMEA2053849	-	n.a.	n.a.	n.a.	n.a.	**2830**	ERR319537
	SAMEA2053913	-	n.a.	n.a.	n.a.	n.a.	**2830**	ERR319550

PhG, phylogroup; ST, sequence-type; n.a., information not available; T, reference strain.

[a] Internal strain collection number of the Biodiversity and Epidemiology of Bacterial Pathogens unit, Institut Pasteur.

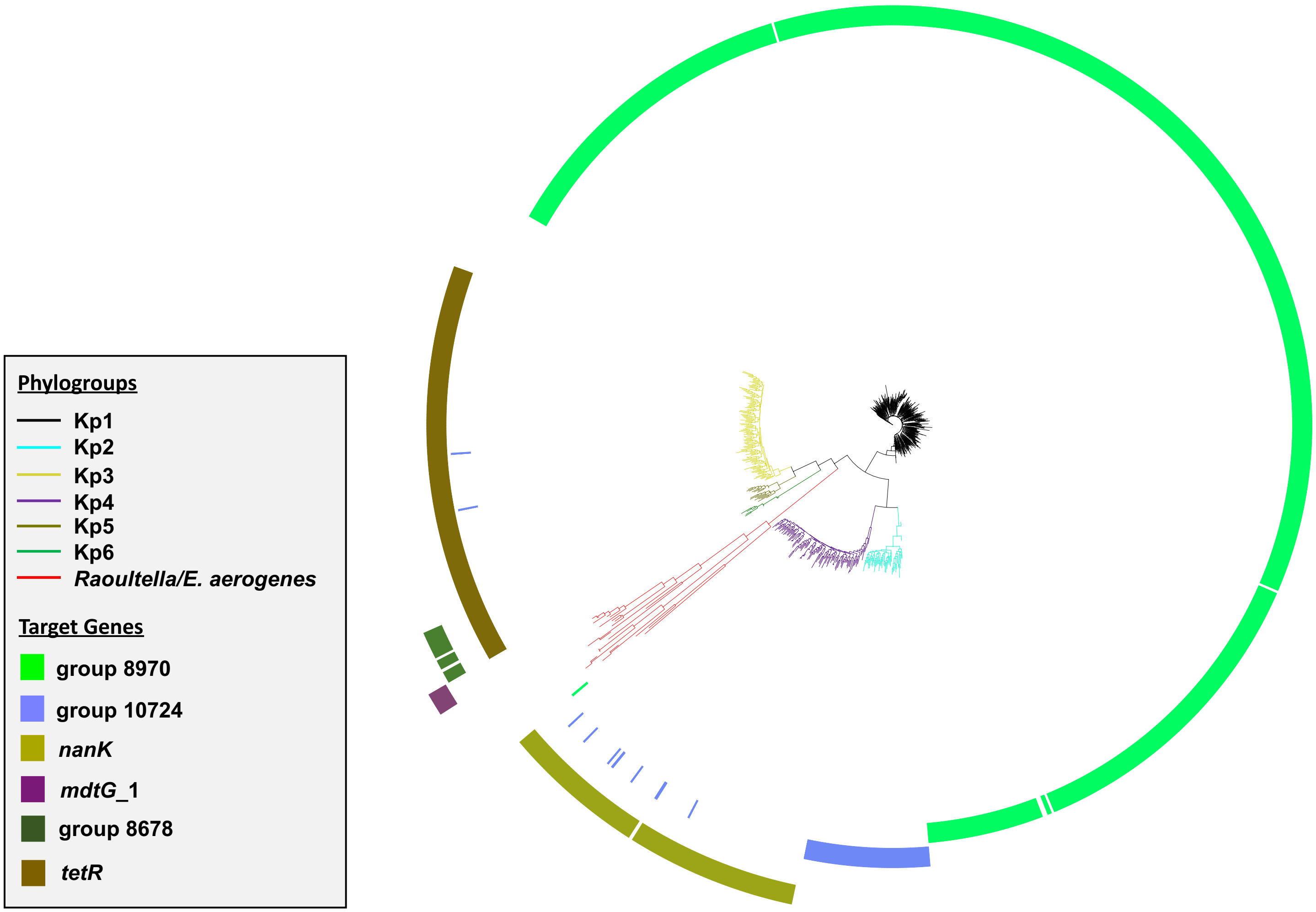

Figure 1. Phylogenetic distribution of the molecular targets selected for the identification of *K. pneumoniae* phylogroups. The branches are colored according to *K. pneumoniae* phylogroups or other closely related species (see color key). Molecular targets were considered as present in the corresponding genomes based on BLASTN, with at least 80% nucleotide identity and 80% length coverage.

Design of primers and probes, and real-time PCR assays

Alignments of the five candidate gene sequences were obtained using Roary. Primers pair and probe targeting each candidate gene were designed using Primer3Plus (http://www.bioinformatics.nl/cgi-bin/primer3plus/primer3plus.cgi) (Table 2) and tested *in silico* using BLASTN on the GenBank nucleotide collection from the NCBI database.

All real-time PCR assays were performed on an ABI StepOne real-time thermocycler (Thermo Fischer Scientific) with the following program: 95°C for 3 min and 40 cycles at 95°C 15 s and 60°C for 1 min. For each assay, DNA was amplified in a 20-µl PCR mix containing 12.5 µl of Takyon Rox PROBE MasterMix 2x (Eurogentec, Belgium), 1 µl of each primer (final concentrations, 500 nM), 0.5 µl of the probe (final concentrations, 250 nM), 2.5 µl of template DNA, and 2.5 µl of PCR-grade water. The specificity of the primers and probes was experimentally tested on 2 ng of purified DNA of representatives of 47 KpSC (phylogroups Kp1 to Kp6) and 20 non-*KpSC* strains (Table 3). Concerning Kp1, Kp2, Kp3/Kp5, Kp4 and Kp6 phylogroup identification, the primers and probe amplified the target sequences in all phylogroup-related strains with a threshold cycle (Ct) value ranging from 18 to 27. In contrast, no amplification was observed on non-targeted strains or with Ct above 37 for few isolates. For Kp7 strains, *in silico* analyses showed that Kp7 reference strain (SB5857T) shared 99.05% identity with the *mdtG1* gene (Kp6 target) and 100% identity in the primer and probe sequences. For the remaining Kp7 public genomes (n=9), the same was not observed, *mdtG1* was not detected (at least using 80% nucleotide identity and 80% length coverage) in the genomic sequence, and none of the remaining four phylogroup defined targets was detected. Kp7 DNA amplification was not tested experimentally and this should be the subject of future evaluation when more Kp7 strains are available.

The corresponding protocol was released publicly on protocols.io (dx.doi.org/10.17504/protocols.io.b4s5qwg6).

Table 2. Primers and probes designed for KpSC phylogroup identification.

Target phylogroup	Target gene	Forward primer	Reverse primer	Probe	Quencher	Reporter	Amplicon length (bp)
**Kp1**	group_8970	CCTCAAACACGCCAATATGC	TACCGCGACGAGTAAAGTGG	GATCCATTGATTCCATTCGAACCGG	BHQ-1	JOE	85
**Kp2**	group_10724	TTGTTGATTGGCAGGCCTTC	AAATGCTGTGACCACCGTTG	CGCCTGGCGTCAGTGGCCCCGAGC	BHQ-1	JOE	70
**Kp3/Kp5**	*tetR*	GCGGGCCGGCTTTTC	CGCATCCCAGGGTATATTCG	AGGCCGAAGCCGCAATGATATTACTCA	BHQ-1	FAM	71
**Kp4**	*nanK*	AAACAGCGGATGCTCTGAAC	ATGAGTCCGCCAAGATTCTG	ATAGCCTCGACCGGGATTATTCGTGA	BHQ-2	TAMRA	145
**Kp6**	*mdtG_1*	GCGTTTCAATGATCCTGTCC	ATCTGCGTCTGGAAAACAG	GTGCGCTGATTGGTATTGGATATGGC	BHQ-2	TAMRA	76

Table 3. Bacterial isolates used to test the specificity of the qPCR assays.

Taxon (Phylogroup)	Strain name	Strain bank (SB) ID*	Country	Isolation year
Klebsiella pneumoniae (Kp1)	SB4-2	**SB1067**	Netherlands	2002
K. pneumoniae (Kp1)	ATCC 13883^{T}	**SB132**	n.a.	n.a.
K. pneumoniae (Kp1)	MGH 78578	**SB107**	n.a.	1994
K. pneumoniae (Kp1)	none	**SB1139**	Netherlands	2002
K. pneumoniae (Kp1)	5-2	**SB617**	Netherlands	2000
K. pneumoniae (Kp1)	04A025	**SB20**	France	1997
K. pneumoniae (Kp1)	2-3	**SB612**	Netherlands	2000
K. pneumoniae (Kp1)	BJ1-GA	**SB4496**	France	2011
K. pneumoniae (Kp1)	MIAE07651	**-**	France	2015
K. quasipneumoniae subsp. *quasipneumoniae* (Kp2)	01A030^{T}	**SB11**	Austria	1997
K. quasipneumoniae subsp. *quasipneumoniae* (Kp2)	none	**SB1124**	Netherlands	2002
K. quasipneumoniae subsp. *quasipneumoniae* (Kp2)	U41	**SB2110**	Germany	1990
K. quasipneumoniae subsp. *quasipneumoniae* (Kp2)	10A442	**SB224**	Italy	1998
K. quasipneumoniae subsp. *quasipneumoniae* (Kp2)	99-1002	**SB2478**	Netherlands	1999
K. quasipneumoniae subsp. *quasipneumoniae* (Kp2)	18A451	**SB255**	Spain	1998
K. quasipneumoniae subsp. *quasipneumoniae* (Kp2)	11128	**SB3445**	n.a.	n.a.
K. quasipneumoniae subsp. *quasipneumoniae* (Kp2)	18A69	**SB59**	Spain	1997
K. quasipneumoniae subsp. *quasipneumoniae* (Kp2)	Kleb Ali 0320584	**SB98**	n.a.	n.a.
K. *variicola* subsp. *variicola* (Kp3)	01A065	**SB1**	Austria	1997

K. *variicola* subsp. *variicola* (Kp3)	07A058	**SB31**	Germany	1997
K. *variicola* subsp. *variicola* (Kp3)	IPEUC-1516	**SB3278**	France	1988
K. *variicola* subsp. *variicola* (Kp3)	CIP 53.24	**SB3295**	n.a.	n.a.
K. *variicola* subsp. *variicola* (Kp3)	Ørskov 1756/51	**SB3301**	n.a.	n.a.
K. *variicola* subsp. *variicola* (Kp3)	F2R9^T	**SB48**	Mexico	n.a.
K. *variicola* subsp. *variicola* (Kp3)	6115	**SB489**	n.a.	n.a.
K. *variicola* subsp. *variicola* (Kp3)	Ørskov 4425/51	**SB497**	n.a.	n.a.
K. *variicola* subsp. *variicola* (Kp3)	Kp342	**SB579**	USA	n.a.
K. *quasipneumoniae* subsp. *similipneumoniae* (Kp4)	09A323	**SB164**	Greece	1997
K. *quasipneumoniae* subsp. *similipneumoniae* (Kp4)	12A476	**SB203**	Netherlands	1998
K. *quasipneumoniae* subsp. *similipneumoniae* (Kp4)	07A044^T	**SB30**	Germany	1997
K. *quasipneumoniae* subsp. *similipneumoniae* (Kp4)	325	**SB3233**	France	1975
K. *quasipneumoniae* subsp. *similipneumoniae* (Kp4)	Ørskov 1303/50	**SB3297**	Turkey	n.a.
K. *quasipneumoniae* subsp. *similipneumoniae* (Kp4)	Ørskov 4463/52	**SB500**	n.a.	n.a
K. *quasipneumoniae* subsp. *similipneumoniae* (Kp4)	CIP 110288	**SB4697**	China	2010
K. *quasipneumoniae* subsp. *similipneumoniae* (Kp4)	1-1	**SB610**	Netherlands	2000
K. variicola subsp. *tropica* (Kp5)	Gal12	**SB824**	Mexico	n.a.
K. variicola subsp. *tropica* (Kp5)	CDC 4241-71	**SB94**	n.a.	n.a.
K. variicola subsp. *tropica* (Kp5)	885	**SB5439**	Madagascar	2016
K. variicola subsp. *tropica* (Kp5)	1266^T	**SB5531**	Madagascar	2016
K. variicola subsp. *tropica* (Kp5)	1283	**SB5544**	Madagascar	2016
K. variicola subsp. *tropica* (Kp5)	1375	**SB5610**	Madagascar	2016

K. variicola subsp. *tropica* (Kp5)	814	**SB5387**	Madagascar	2015
'K. quasivariicola' (Kp6)	08A119	**SB33**	Germany	1997
'K. quasivariicola' (Kp6)	10982	**SB6071**	USA	2005
'K. quasivariicola' (Kp6)	01-467-2ECBU	**SB6094**	Madagascar	2015
'K. quasivariicola' (Kp6)	01-310A	**SB6095**	Madagascar	2013
'K. quasivariicola' (Kp6)	KPN1705^{T}	**SB6096**	USA	2014
K. michiganensis (Ko1)	CIP 110787^{T}	**SB4934**	USA	2010
K. michiganensis (Ko1)	05A071	**SB71**	France	1997
K. michiganensis (Ko1)	09A029	**SB78**	Greece	1997
K. grimontii (Ko6)	07A479	**SB324**	Germany	1998
K. grimontii (Ko6)	06D090	**SB352**	France	1998
K. grimontii (Ko6)	06D021^{T}	**SB73**	France	1997
K. oxytoca (Ko2)	ATCC 13182^{T}	**SB175**	n.a.	n.a.
K. oxytoca (Ko2)	02A067	**SB131**	Belgium	1997
K. oxytoca (Ko2)	NCTC 49131	**SB136**	n.a	n.a
K. aerogenes	MIAE07652	**-**	France	n.a.
K. aerogenes	CIP 60.86^{T}	**SB3629**	France	n.a.
K. aerogenes	01A089	**SB538**	Austria	1997
K. aerogenes	02A002	**SB539**	Belgium	1997
Raoultella terrigena	ATCC 33257^{T}	**SB170**	n.a.	n.a.
R. terrigena	17C143	**SB313**	Spain	1998
R. terrigena	V9813596	**SB2796**	Netherlands	1998
R. planticola	01A041	**SB7**	Austria	1997
R. planticola	ATCC 33531^{T}	**SB174**	n.a.	n.a.
R. planticola	12C169	**SB303**	Netherlands	1998
R. ornithinolytica	ATCC 31898	**SB171**	n.a.	n.a.

n.a., information not available; T, reference strain.

* Internal strain collection number of the Biodiversity and Epidemiology of Bacterial Pathogens unit, Institut Pasteur.

References

1. Page AJ, Cummins CA, Hunt M, Wong VK, Reuter S, Holden MT, Fookes M, Falush D, Keane JA, Parkhill J. 2015. Roary: rapid large-scale prokaryote pan genome analysis. Bioinformatics 31:3691-3. doi:10.1093/bioinformatics/btv421.
2. Barbier E, Rodrigues C, Depret G, Passet V, Gal L, Piveteau P, Brisse S. 2020. The ZKIR Assay, a Real-Time PCR Method for the Detection of Klebsiella pneumoniae and Closely Related Species in Environmental Samples. Appl Environ Microbiol 86. doi:10.1128/AEM.02711-19.
3. Criscuolo A. 2019. A fast alignment-free bioinformatics procedure to infer accurate distance-based phylogenetic trees from genome assemblies. Research Ideas and Outcome 5:e36178.
4. Letunic I, Bork P. 2021. Interactive Tree Of Life (iTOL) v5: an online tool for phylogenetic tree display and annotation. Nucleic Acids Res 49:W293-W296. doi:10.1093/nar/gkab301.
